# Cataloging Human *PRDM9* Allelic Variation Using Long-Read Sequencing Reveals *PRDM9* Population Specificity and Two Distinct Groupings of Related Alleles

**DOI:** 10.3389/fcell.2021.675286

**Published:** 2021-11-04

**Authors:** Benjamin Alleva, Kevin Brick, Florencia Pratto, Mini Huang, Rafael Daniel Camerini-Otero

**Affiliations:** Genetics and Biochemistry Branch, National Institute of Diabetes and Digestive and Kidney Diseases, National Institutes of Health, Bethesda, MD, United States

**Keywords:** *PRDM9*, meiosis, recombination, human, long-read sequencing, minisatellite genotyping

## Abstract

The PRDM9 protein determines sites of meiotic recombination in humans by directing meiotic DNA double-strand breaks to specific loci. Targeting specificity is encoded by a long array of C_2_H_2_ zinc fingers that bind to DNA. This zinc finger array is hypervariable, and the resulting alleles each have a potentially different DNA binding preference. The assessment of *PRDM9* diversity is important for understanding the complexity of human population genetics, inheritance linkage patterns, and predisposition to genetic disease. Due to the repetitive nature of the *PRDM9* zinc finger array, the large-scale sequencing of human *PRDM9* is challenging. We, therefore, developed a long-read sequencing strategy to infer the diploid *PRDM9* zinc finger array genotype in a high-throughput manner. From an unbiased study of *PRDM9* allelic diversity in 720 individuals from seven human populations, we detected 69 *PRDM9* alleles. Several alleles differ in frequency among human populations, and 32 alleles had not been identified by previous studies, which were heavily biased to European populations. *PRDM9* alleles are distinguished by their DNA binding site preferences and fall into two major categories related to the most common *PRDM9-A* and *PRDM9-C* alleles. We also found that it is likely that inter-conversion between allele types is rare. By mapping meiotic double-strand breaks (DSBs) in the testis, we found that small variations in *PRDM9* can substantially alter the meiotic recombination landscape, demonstrating that minor *PRDM9* variants may play an under-appreciated role in shaping patterns of human recombination. In summary, our data greatly expands knowledge of *PRDM9* diversity in humans.

## Introduction

Meiosis is a specialized cellular division that creates gametes. During meiosis, hundreds of programmed DNA double-strand breaks (DSBs) are formed and repaired *via* specialized pathways: these pathways assure proper chromosome segregation and introduce genetic diversity through the exchange of genetic information between parental chromosomes. In humans and many other mammals, meiotic DSB localization is defined by the DNA binding specificity of the meiosis-specific PRDM9 protein, which creates DSB hotspots (Hayashi et al., 2005; Baudat et al., 2010; Myers et al., 2010; Parvanov et al., 2010). PRDM9 is composed of four functional domains: KRAB and SSXRD domains play an unknown role but are thought to mediate protein–protein interactions (Imai et al., 2017; Parvanov et al., 2017; Thibault-Sennett et al., 2018), a PR/SET domain with histone methyltransferase activity (Wu et al., 2013; Koh-Stenta et al., 2014), and an array of C_2_H_2_ zinc fingers (ZFs) that confer DNA binding specificity (Baudat et al., 2010; Grey et al., 2011; Billings et al., 2013; Koh-Stenta et al., 2014; Walker et al., 2015).

Since hotspot loci are targeted for recombination by PRDM9, gene conversion and mutation during DNA repair rapidly erodes *PRDM9* binding sites in the genome. Thus, the emergence of new alleles is favored as a means of “escaping” the detrimental effects of binding site depletion (Myers et al., 2010; Lesecque et al., 2014). The *PRDM9* C_2_H_2_ ZF array is under strong positive selection (Oliver et al., 2009; Buard et al., 2014; Schwartz et al., 2014; Ahlawat et al., 2016) and, as a result, is hypervariable in all species studied to date with a full-length *PRDM9* gene. Currently, 33 *PRDM9* alleles (from here on, *PRDM9* alleles are defined as the sequence variation found within the ZF array) have been identified in human population studies (Berg et al., 2010, 2011), dozens of alleles in apes (Auton et al., 2012; Schwartz et al., 2014), and >170 alleles in mice (Buard et al., 2014; Kono et al., 2014). In addition, hundreds of alleles have been identified in human sperm (Jeffreys et al., 2013). The mechanisms that give rise to *PRDM9* variation remain opaque; however, alleles can differ by the number of ZFs, combinations of ZFs, or even by a single nucleotide. It is important to understand variation at this locus since different *PRDM9* alleles can completely alter the recombination landscape by altering the preferred DNA binding site (Baudat et al., 2010; Brick et al., 2012; Pratto et al., 2014b; Smagulova et al., 2016). The distribution of *PRDM9* alleles differs among human populations, with by far the greatest diversity of *PRDM9* alleles is found in Africa (Hinch et al., 2011). Non-African populations are dominated by a single *PRDM9* allele (*PRDM9-A*); for example, in populations of European origin, the *PRDM9-A* allele was found to be present at a frequency > 80%. The *A* allele is also highly prevalent in African populations (∼50%), and its prevalence outside of Africa may stem from a historical genetic bottleneck. In contrast, the next most frequent allele, *PRDM9-C*, is far more frequent in African (∼15% frequency) than in European populations (∼1% frequency) [data from Baudat et al. (2010), Berg et al. (2010), and Parvanov et al. (2010)].

Despite these clear differences among populations, extant studies of *PRDM9* allelic diversity disproportionately surveyed individuals of European descent [628/750 individuals; data from Baudat et al. (2010), Berg et al. (2010), and Parvanov et al. (2010)], and a comprehensive survey of *PRDM9* alleles across human populations has never been performed. The catalog of human genetic diversity has enormously expanded in recent years through whole-genome sequencing and exome sequencing of individual genomes. However, the short-read technology used for these advances is not suited for sequencing the highly repetitive *PRDM9* ZF array, which still relies on labor-intensive Sanger sequencing. In this study, we developed a high-throughput long-read sequencing-based approach to determine the diploid *PRDM9* genotype of 720 individuals from seven distinct human populations. We identified 32 previously unannotated alleles and found that the prevalence of some *PRDM9* alleles differs substantially between populations. Additionally, we identified single-nucleotide polymorphisms (SNPs) associated with different *PRDM9* genotypes. We also demonstrate that although most human *PRDM9* alleles are related to either the *PRDM9-A* or *PRDM9-C* alleles, even superficially minor changes to the *PRDM9* ZF array can substantially re-shape the recombination landscape.

## Results

### Human Populations Surveyed for *PRDM9* Genotyping

**FIGURE 1:**
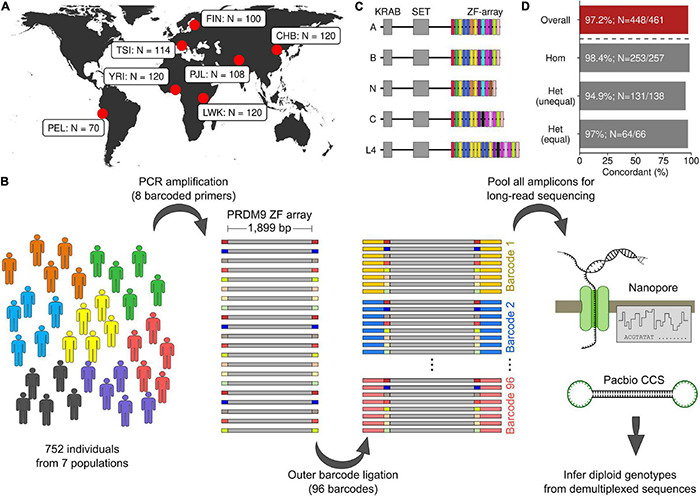
*PRDM9* diploid genotyping with long-read sequencing. **(A)** Geographic location of seven populations in this study (YRI, Yoruba in Ibadan, Nigeria; LWK, Luhya in Webuye, Kenya; TSI, Toscani in Italia; FIN, Finnish in Finland; PEL, Peruvian in Lima, Peru; CHB, Han Chinese in Beijing, China; PJL, Punjabi in Lahore, Pakistan). **(B)** Schematic of amplification, barcoding, and sequencing strategy (see section “Materials and Methods”). **(C)** The protein domain structure of human PRDM9. The zinc finger (ZF) array of *PRDM9* is a repeating array of 84-bp-long ZFs. ZF variants are indicated by different colors. Five annotated *PRDM9* alleles are shown. **(D)** Genotyping *PRDM9* gives analogous results using either Oxford Nanopore or Pacific Biosciences Circular Consensus Sequencing (CCS) (PacBio CCS). The percentage of concordant genotypes is shown. The least agreement (94.9%) is seen for individuals that are heterozygous for alleles of different lengths [Het (unequal)]. The overall concordance across all 461 individuals is 97.2%.

Fine-scale recombination maps differ among human populations, which may represent differences in the distribution of *PRDM9* alleles (Spence and Song, 2019). Recombination maps broadly cluster into five geographic groups (European, African, East Asian, South Asian, and South American; Spence and Song, 2019); therefore, we assessed the diversity of *PRDM9* alleles in at least one representative population from each cluster (Figure 1A). Most studies of *PRDM9* diversity in humans have been performed in individuals of European descent, so to assess if *PRDM9* diversity differed among European ethnic groups, we chose two European populations—one with little admixture (Finnish in Finland; FIN) and one with more admixture (Toscani in Italia, TSI; Zaidi et al., 2019). A few studies previously addressed *PRDM9* diversity in Asian populations; therefore, we chose two Asian populations for study; one from East Asia (Han Chinese in Beijing, CHB) and one from South Asia (Punjabi in Lahore, Pakistan, PJL). African populations have a high diversity of *PRDM9* alleles (Berg et al., 2011; Hinch et al., 2011); to assess similarities and differences in the *PRDM9* repertoire among African populations, we chose to survey *PRDM9* diversity in the Yoruba in Ibadan, Nigeria (YRI), and in the Luhya in Webuye, Kenya (LWK). Finally, we chose a South American population as no prior studies have examined *PRDM9* diversity in this geographic region (Peruvian in Lima, Peru, PEL). For each population, we attempted to infer the diploid *PRDM9* genotype for all individuals.

### Long-Read Sequencing of Human *PRDM9*

To analyze *PRDM9* allelic diversity in seven different populations, we devised a workflow to amplify and sequence the *PRDM9* ZF array using long-read sequencing (see section “Materials and Methods”; Figures 1B,C). We amplified the *PRDM9* ZF array from the genomic DNA of each individual using PCR primers containing one of eight unique DNA barcodes. Samples were subsequently pooled in sets of eight, and a second barcode was added using one of the 96 barcodes from the Oxford Nanopore PCR Barcoding Kit 1-96. All barcoded amplicons were then pooled for long-read sequencing.

The repetitive *PRDM9* ZF array causes PCR amplification artifacts that are seen as laddering and smearing in gel electrophoresis images (Supplementary Figure S1; see also Schwartz et al., 2014). Previous approaches for defining *PRDM9* alleles using Sanger sequencing required manual excision of the desired band. Instead, we removed amplification artifacts later, *in silico*, by retaining only reads with an uninterrupted and contiguous array of ZFs signified by the presence of the expected genomic flanking sequences (see section “Materials and Methods”). Amplification errors may also create reads with an erroneous, but complete, ZF array. Although these will pass the initial filter, they represent a minority of reads (Supplementary Figure S1) and will only negligibly affect consensus-based genotyping.

To compare the utility of different long-read sequencing platforms for *PRDM9* genotyping, we inferred the *PRDM9* diploid genotype for 461 individuals using both Oxford Nanopore sequencing and PacBio Circular Consensus Sequencing (CCS). For 97.2% of individuals, the inferred diploid *PRDM9* genotype agreed using both platforms (448/461; Figure 1D). In 12/13 individuals with discordant genotypes, at least one allele was identified in both datasets (Supplementary Figures S2A–C); thus, the absolute error rate of genotype calls is ∼1.5% (14/922 alleles). The highest agreement was in individuals homozygous for a *PRDM9* genotype, where genotyping is least challenging (98.4% agreement; Figure 1D). Individuals heterozygous for *PRDM9* but where both *PRDM9* alleles had the same number of ZFs are theoretically the most challenging to accurately genotype, as the *PRDM9* alleles can differ by as little as a single nucleotide. However, 97.0% of diploid genotypes agreed across platforms (Figure 1D). Somewhat surprisingly, the agreement was lowest for individuals who were heterozygous for *PRDM9* but where the inferred *PRDM9* alleles had differing numbers of ZFs (94.9% agreement; Figure 1D). These discrepancies were likely due to samples with low coverage from one sequencing technology or samples with artifacts from PCR that became overrepresented during sequencing and data processing (Supplementary Figures S2A–C). Given the extensive concordance, we merged nanopore and CCS reads for final genotype calling (see section “Materials and Methods”). To assess the final accuracy, we examined *PRDM9* diversity in trios. For 31/32 YRI individuals with both parents in the YRI population, the diploid *PRDM9* genotype was concordant with the parental genotypes. 63/64 alleles were consistent with the parents, alluding to an overall error rate of ∼1.6%. This is very close to the genotyping error rate inferred by comparing sequencing technologies (∼1.5%). The diploid *PRDM9* genotype was also correctly identified in two control samples where *PRDM9* was independently determined using Sanger sequencing (CTL: Supplementary Figures S1C,D: lanes 6 and 8). Ultimately, we identified the diploid *PRDM9* genotype for 720/752 individuals within the seven different populations. The remaining individuals lacked sufficient coverage depth for genotyping (Supplementary Figures S2E–G).

### A Catalog of *PRDM9* Diversity in Humans

**FIGURE 2:**
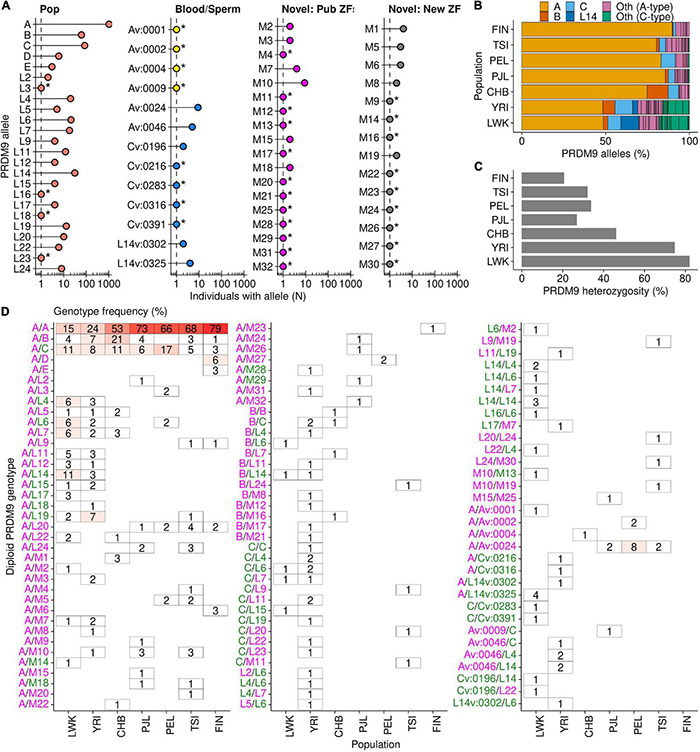
*PRDM9* diploid genotypes in seven populations. Sixty-nine total *PRDM9* alleles were found in 720 individuals. **(A)** Approximately half of the *PRDM9* alleles sequenced in this study are found in >1 individual [asterisk (*) denotes alleles found in one individual]. Alleles are split by type; Pop (red) = *PRDM9* allele found in previous population studies; Sperm/Blood = found as a *PRDM9* variant in blood (yellow) or sperm (blue); Novel: Pub ZFs (pink) = novel *PRDM9* allele identified in this study and contains only known ZFs; Novel: New ZF (gray) = novel *PRDM9* allele identified in this study and contains at least one new ZF. **(B)** Composition of *PRDM9* alleles in each population. The four most prevalent alleles are color coded (*A*—yellow; *B*—orange; *C*—light blue; *L14*—dark blue). All other alleles are color coded by *A*-type or *C-type* allele (described in Supplementary Figure S4; *A*-type = magenta and *C*-type = green). **(C)** Frequency of *PRDM9* heterozygosity in each population. **(D)** Frequency of diploid *PRDM9* genotypes in each population. Blank spaces indicate genotypes not found. *A*-type allele labels are magenta and *C*-type are green.

We identified 69 different *PRDM9* alleles among 720 individuals for whom we could infer the diploid genotype; 24 of these alleles had been previously identified in human population studies (Baudat et al., 2010; Berg et al., 2010, 2011), 13 alleles were previously identified only in human blood (*N* = 4) or sperm (*N* = 9) (Jeffreys et al., 2013), and 32 novel *PRDM9* alleles were identified (Figure 2A). Although our approach may yield spurious “new” alleles (if genotyping/sequencing errors create what appears to be a new ZF, and hence a new allele), we found that a majority of novel alleles (30/32) have secondary support. Alleles derived from new combinations of known ZFs were unlikely to have occurred erroneously and were considered “high confidence” novel alleles (*N* = 18). Five alleles with a novel ZF were found in more than one individual and also likely represent “high confidence” novel alleles. Finally, short-read exome sequencing data from the 1,000 Genomes Project validated seven of the nine remaining alleles (see section “Materials and Methods”; Supplementary Figure S3). The remaining two alleles (*M22* and *M23*) lacked sufficient exome sequencing data to validate, or invalidate, the allele. Given the accuracy of other novel genotypes, it seems unlikely that these are incorrect.

We next examined the predicted binding sites for all human *PRDM9* alleles. Consistent with previous work (Berg et al., 2011; Hinch et al., 2011), we found that *PRDM9* alleles broadly cluster into two groups; those with a *PRDM9-A-type* predicted binding site (*A*-type) and those with a *PRDM9-C-type* predicted binding site (*C*-type) (Supplementary Figure S4A). To formally categorize each allele as either *A*-type or *C*-type and to avoid complexities associated with predicting *PRDM9* binding, we scored each allele by the similarity to the DNA contact residues of the *PRDM9-A* and *PRDM9-C* DNA binding sites (see section “Materials and Methods”; Supplementary Figure S4). By this criteria, 50/71 alleles in our study were *A-type* and 21/71 were *C-type* (note that this includes two alleles found in control experiments but not part of the population analysis—*L13* and *Av:0053*; for allele nomenclature, see section “Materials and Methods”). We found some alleles are quite dissimilar to both (*L5*, *M12*, and *Cv:0283* alleles; Supplementary Figure S4B). 27 *A-type* alleles (not including *PRDM9-A*) had no predicted variation at the DNA contact residues, implying that these alleles likely bind the same DNA sequence as *PRDM9-A*. Likewise, 13 *C-type* alleles (not including *PRDM9-C*) had an identical predicted DNA contact site as *PRDM9-C. A-type* alleles were present in all populations with similar prevalence; however, *C*-type alleles were almost exclusively found in the two African populations (LWK and YRI; Figure 2B).

The length of the *PRDM9* ZF array has been used as a proxy for studying different variants of *PRDM9* (Kong et al., 2010). We found a significant difference between the length of *A*-type and *C*-type alleles (*A*-type median = 13 ZFs, *C*-type median = 15 ZFs; *P* = 10^–5^, Wilcoxon test; Supplementary Figure S4D). *PRDM9* variants that arise in sperm tend to remain similar in size to the allele from which they are derived (Supplementary Figure S4E). Thus, it appears likely that these differences are not shaped by selection in favor of particular allele lengths, but by limitations of the mechanism by which they arise.

### Population Frequency of *PRDM9* Alleles in Seven Human Populations

Consistent with previous studies (Baudat et al., 2010; Berg et al., 2010; Parvanov et al., 2010), we found that, by far, the most frequent *PRDM9* variant in human populations was the *A* allele (Figure 2B and Supplementary Figure S5A). The proportion of *A* alleles was highest in the Finnish population [frequency (fA_*FIN*_) = 90%] and lowest in the two African populations (fA_*LWK*_ = 49% and fA_*YRI*_ = 48%). The Han Chinese population had an intermediate *A* allele frequency (fA_*CHB*_ = 75%), although it is not clear if this differs from the frequency in the other non-African populations (Supplementary Figure S6). Three other alleles (*B*, *C*, and *L14*) were found in ≥10% of individuals in at least one population, and each allele displayed population-specific differences in its distribution. Previously, the *B* allele was found at low frequencies in European and African individuals (2 and 3%, respectively; Berg et al., 2010). Our data paint a different picture of the distribution of this allele. We found that the *B* allele was enriched in the CHB (fB_*CHB*_ = 13%) and YRI populations (fB_*YRI*_ = 7%), compared to the low frequencies in other populations (0–3%; Figure 2B and Supplementary Figures S5A, S6). The prevalence in the CHB population was far more than expected from sampling noise, suggesting that the *B* allele has proliferated substantially in the Han Chinese population compared to others (Supplementary Figure S6). Another example of a population-enriched allele was the *L14* allele, found predominantly in the LWK population (fL14_*LWK*_ = 11%). *L14* was also found in the YRI population, but at a substantially reduced frequency (fL14_*YRI*_ = 3%; Supplementary Figure S5A, S6), and it was absent from the other five (non-African) populations. Finally, the last allele among this tier of alleles was the *C* allele, previously described as the most common minor allele in Africans (Berg et al., 2010). Our data showed that while the *C* allele was indeed relatively frequent in both African populations (fC_*YRI*_ = 10%; fC_*LWK*_ = 8%), it was found at a similar frequency in some non-African populations (fC_*PEL*_ = 8%; fC_*CHB*_ = 6%). The frequency of the *C* allele in the TSI and PJL populations was lower (fC_*TSI*_ = 4%; fC_*PJL*_ = 4%), but within the expected range of sampling error for the YRI, LWK, PEL, and CHB populations (99% C.I.; Supplementary Figure S6). The Finnish population was the major outlier as the *C* allele occurred at just 2% frequency. Together, these data suggest that rather than being an African-enriched allele, the *C* allele is rare in some European populations.

The remaining tier of alleles was present at a frequency of <10% in all populations. Although individually rare, together, these alleles represent 16% of all *PRDM9* alleles (*N* = 230/1,440). The prevalence of these rarer alleles varied by population, and consistent with previous data, rare alleles were most frequent in both African populations (fRare_*LWK*_ = 30%; fRare_*YRI*_ = 31%). The TSI population had the next highest frequency of rare *PRDM9* alleles (fRare_*TSI*_ = 14%), which may have arisen from geographical proximity to Africa and recent admixture. All other populations had relatively similar levels of rare *PRDM9* alleles (fRare_*PJL*_ = 9%; fRare_*PEL*_ = 8%; fRare_*FIN*_ = 8%; fRare_*CHB*_ = 7%). Among the rare alleles were 13 alleles previously only seen as *de novo* variants in blood or sperm (Jeffreys et al., 2013). Six of these alleles were derived from *de novo* variation of *PRDM9-A*, five from *PRDM9-C*, and two from *PRDM9-L14*. Indeed, the population distribution of the variant alleles broadly paralleled that of the alleles from which they were likely derived (Supplementary Figures S5A,B). These findings imply that a previous catalog of several hundred *PRDM9* variants from male meiosis (Jeffreys et al., 2013) represents many *PRDM9* alleles likely present in humans.

Our limited sample size coupled with the rarity of these alleles made it difficult to infer population differences; however, several rare alleles were sufficiently strongly enriched to make some conclusions (Supplementary Figure S6). The *L4*, *L6*, *L7*, *L11*, *L19*, *and Av:0046* alleles were each enriched in at least one African population. Of those alleles, *L6*, *L7*, and *L19* were also found infrequently in at least one non-African population. Two rare alleles (*L20* and *L24*) were enriched in the TSI population and not found in either African population. One rare allele that was previously only found as an *A*-derived variant in blood (*Av:0024*) was enriched in the PEL population, infrequent in two other populations (TSI and PJL), and absent from either African population. Additionally, a novel allele, *M1*, was enriched in the CHB population and absent from all other populations. It is important to note that alleles absent from a population in our study may still be present at a low frequency, below our detection threshold. Perhaps the most intriguing of the rare variants was the *D*-allele. *PRDM9-D* is a so-called “de-stabilizing” allele that appears to cause elevated variation of the ZF array in sperm (Jeffreys et al., 2013). *PRDM9-D* was exclusively found in six individuals in the Finnish population (Supplementary Figure S5) and is therefore a strong candidate for a population-enriched allele outside of Africa (Supplementary Figure S6).

### Diploid *PRDM9* Genotypes in 720 Individuals

Importantly, and in contrast to previous studies of human *PRDM9* diversity, our approach analyzed the inferred phased diploid *PRDM9* genotype for each individual. Knowledge of diploid genotypes is important because *PRDM9* heterozygosity alters the recombination landscape (Pratto et al., 2014b), allelic dominance can alter the contribution of each *PRDM9* allele (Brick et al., 2012; Pratto et al., 2014b; Davies et al., 2016; Smagulova et al., 2016), and genetic incompatibilities in *Prdm9* heterozygotes can cause male sterility (in mice, Mihola et al., 2009; Flachs et al., 2012, 2014; Smagulova et al., 2016; Kusari et al., 2020; Mukaj et al., 2020). We found that the prevalence of *PRDM9* heterozygosity was directly proportionate to the frequency of the most prevalent alleles (Figure 2C and Supplementary Figure S5). Thus, far more individuals were heterozygous for *PRDM9* in populations where the *PRDM9-A* allele was less prevalent and where allelic diversity was the highest (LWK and YRI populations; 82 and 75%, respectively; Figures 2C,D and Supplementary Figure S5). Interestingly, the CHB population had the third highest level of heterozygosity even though it also had relatively low *PRDM9* allelic diversity (10 alleles in the population). This is likely due to the relatively high prevalence of the *PRDM9-B* allele.

### Sequence Polymorphisms Associate With *PRDM9* Genotype

A single haplotype, encompassing *PRDM9* and characterized by the rs6889665 SNP, was shown to be strongly associated with differences in the recombination landscape between Europeans and Africans (Hinch et al., 2011). Another SNP (rs2914276) was associated with alterations to the recombination landscape in the Icelandic population (Kong et al., 2010). The *PRDM9* genotypes of individuals were unknown in these previous works; however, the implication is that *PRDM9* alleles may be associated with different haplotypes in humans.

**FIGURE 3:**
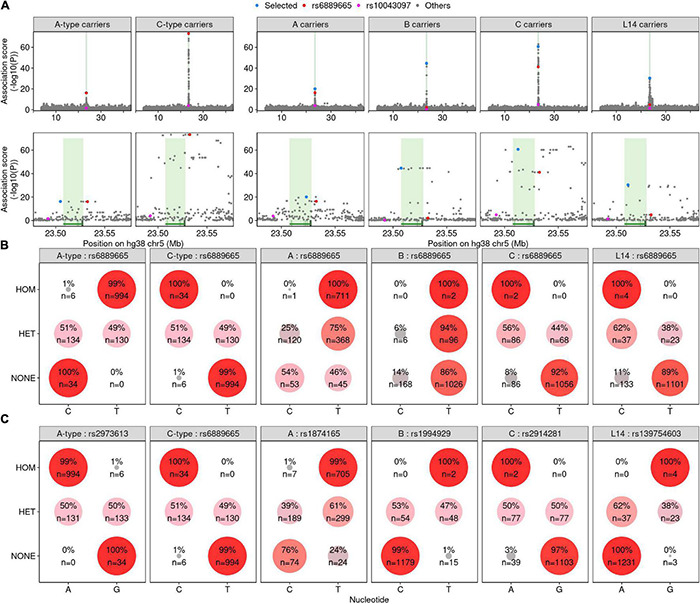
Single-nucleotide polymorphisms (SNPs) are associated with different *PRDM9* alleles. **(A)** Association scores for all SNPs in a ±20-Mb window around the *PRDM9* locus (see “Materials and Methods”). The *PRDM9* genotype examined is given in the title of each panel (individuals carrying at least one copy of *A*-type, *C*-type, *A*, *B*, C, or *L14 PRDM9* alleles, respectively). SNPs previously shown to be linked to recombination patterns in African populations (Hinch et al., 2011) are marked in red (rs6889665) and pink (rs10043097). Lower panels show a magnified view around the peak in the association signal at *PRDM9*. The *PRDM9* gene is indicated by green shading. **(B)** Assessment of the prevalence of rs6889665 alleles. Individuals were classified as homozygous (HOM), heterozygous (HET), or non-carriers (NONE) of the *PRDM9* allele indicated in gray in the column header. The prevalence of the C and T alleles of rs6889665 were assessed in each group. Larger circle size and deeper red color indicate a higher prevalence. **(C)** Similar to **(B)**, but for the best-scoring SNP [blue in panel **(A)**] for each *PRDM9* genotype we tested.

To first approximate the associations found in Hinch et al. (2011), where the prevalence of *PRDM9-C*-type alleles was likely the major contributor to differences between African and European-derived recombination maps, we examined SNPs that broadly associated with *A*-type or *C*-type *PRDM9* allele carriers (Figure 3). Consistent with Hinch et al. (2011), rs6889665 was among the strongest associated SNPs for both groups (Figure 3A). We next performed more specific association tests for the *A*, *B*, *C*, and *L14* alleles of *PRDM9*; these were the most frequent alleles found in our study, and we identified at least one homozygous individual for each. For all four alleles, we found strong evidence of an associated haplotype in a narrow region around the *PRDM9* gene (Figure 3A). rs6889665 was associated with the *PRDM9*-*A* and *PRDM9*-*C* alleles; however, it was not the most strongly associated SNP for either allele. In addition, the rs6889665 polymorphisms did not associate with all alleles; for example, it was not associated with *PRDM9*-*B* (*A*-type allele). Therefore, we assessed the prevalence of each haplotype by examining the most highly associated SNP for each allele. The T and C alleles of rs6889665 were strongly enriched in individuals with the *PRDM9-A* and *PRDM9-C* alleles, respectively (Figure 3B). However, other SNPs associated with *PRDM9*-*A* (rs1874165) and *PRDM9-C* (rs2914281) exhibited more pronounced enrichment (Figure 3C). Thus, rs6889665 did not appear to be associated with a single *PRDM9* allele, but rather with *A*-type/*C*-type groups of alleles. For example, the best hit for the *PRDM9-A*-associated SNP (rs1874165) was the T allele of rs1874165, which was present in 99% of *PRDM9-A* homozygotes (Figure 3C). The T allele of rs1874165 was also present in 24% of individuals without the *PRDM9-A* allele. However, all these individuals had a *PRDM9-A*-type allele and the T allele of rs1874165 was never found in individuals that lack a *PRDM9-A*-type allele. In contrast, the T allele of rs1994929, the A allele of rs2914281, and the G allele of rs139754603 almost exclusively occurred in association with *PRDM9-B*, *PRDM9*-C, and *PRDM9-L14*, respectively (Figure 3C). Individuals carrying these SNP alleles but not the associated *PRDM9* allele were enriched in the populations where each allele was most prevalent (Supplementary Figure S7). Thus, these SNPs may also segregate with similar *PRDM9* variants and may exhibit population specificity. The *L14*-associated variant (G allele of rs139754603) was rarely found in *PRDM9*-*C* carriers (1/148 alleles in individuals that did not have *PRDM9-L14*), despite *PRDM9-L14* being a *C*-type allele with a fully intact predicted *PRDM9*-*C* binding site. Similarly, the *PRDM9-C*-associated haplotype (A allele of rs2914281) was rare in *PRDM9*-*L14* carriers (2/44 alleles in individuals that did not have *PRDM9-C*).

### Isolated Clusters of *A*-Type and *C*-Type *PRDM9* Alleles in Humans

The repeating 84-bp sequences that make up the *PRDM9* ZF array constitute a minisatellite-like structure. Minisatellites are known to be hotspots of genome instability, which may mediate the appearance of new *PRDM9* alleles. The mechanisms underlying minisatellite instability remain opaque, making relatedness between *PRDM9* alleles difficult to infer; however, empirical observations demonstrate that template switches at minisatellites (mediated either *via* replicative errors or gene conversion) can explain the expansion and contraction of minisatellite arrays (Jurka and Gentles, 2006). A previous study that examined the formation of novel *PRDM9* alleles in human blood and sperm suggested that the formation of new *PRDM9* alleles is due to template switching during replication and/or repair in mitotic and meiotic cells (Jeffreys et al., 2013).

To explore potential relatedness among alleles, we developed an algorithm to simulate putative template switching events between *PRDM9* alleles (parental alleles) that may result in the formation of another allele (child allele) (see section “Materials and Methods”; Supplementary Figure S8). Our approach is agnostic to the mechanism by which template switching occurs.

**FIGURE 4:**
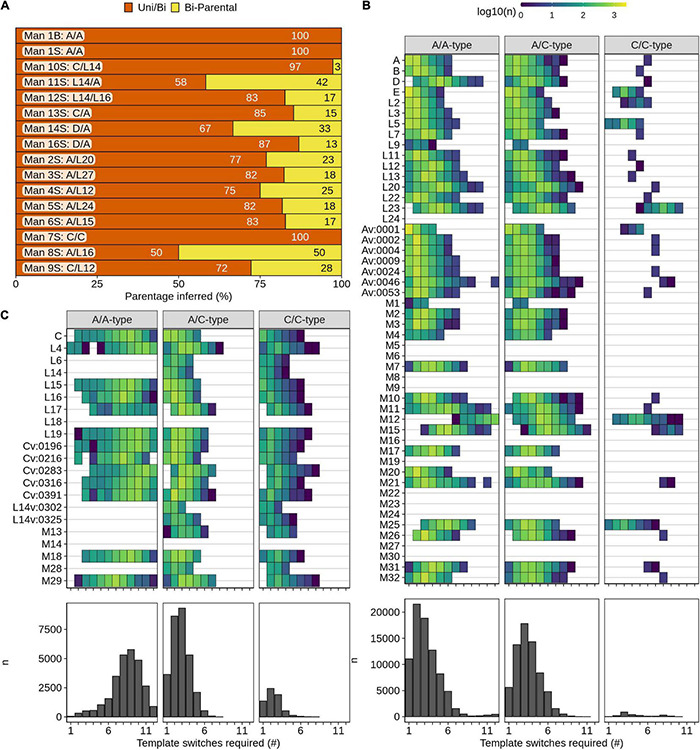
Isolated clusters of *A*-type and *C*-type *PRDM9* alleles. The *PRDM9* ZF array is hypervariable, and variation arises *via* a poorly understood mechanism. We designed and utilized an algorithm to predict the formation of any *PRDM9* allele from any other based on template switching. **(A)** Most *PRDM9* variants in human blood or sperm can be explained by template switches between the two parental *PRDM9* alleles. If several combinations of parental alleles are possible, we identified the “most-likely” recombinant, which required the minimal number of template switches. For each individual, we quantified the alleles where this “most-likely” recombinant is derived from either both parental alleles (bi-parental) or where a uni- and bi-parental origin are equally possible (Uni/Bi). **(B)** *A*-type *PRDM9* alleles rarely arise from *C*-type alleles, and **(C)** *C*-type *PRDM9* alleles can arise from *A*-type alleles but mostly require highly complex template switches. **(B,C)** We searched for potential parental alleles for each *A*-type **(B)** and *C*-type **(C)** *PRDM9* allele. All alleles found in human populations or in blood/sperm only were considered. Heatmaps show the number of potential parental combinations for each number of template switches. Columns represent events in *A*-type homozygotes, *A*-type/*C*-type heterozygotes, or *C*-type homozygotes. Quantitation of all events is shown in bar plots underneath.

We first examined the *PRDM9* variants that were documented in the sperm and blood of individuals where the parental alleles were known (Jeffreys et al., 2013). We found that all variants could be explained by template switching (Figure 4A and Supplementary Figure S9). Consistent with previous findings, *PRDM9* variants from the blood all derived from a single template switch, whereas variants in sperm often required complex events with >1 switch (Supplementary Figure S9; Jeffreys et al., 2013). Most sperm-derived variants could be formed from interactions involving either one or both parental alleles. Intriguingly, in men heterozygous for *PRDM9*, approximately a quarter of all sperm-derived variants required template switching between the two parental alleles and could not be derived from just one parental allele. The percentage of such alleles was highest for men with one *A*-type and one *C*-type allele (Man8—50%, Man11—42%), where inter-homolog switches were less likely to be masked by similarities between parental alleles. This implies that inter-homolog template switches are a major mechanism by which *PRDM9* variants are generated in the germline.

We next applied our algorithm to assess which *PRDM9* alleles in the human population could be derived from others. 38/50 *A*-type alleles (Figure 4B) and 19/21 *C*-type alleles (Figure 4C) could be derived from other annotated human *PRDM9* alleles *via* template switching. 12/14 *PRDM9* alleles that could not be derived from others were novel alleles found in this study (e.g., *M5, M6, M8, M14*, etc.). Novel alleles are likely over-represented because they lack parental representation in the population, or their parental alleles may be extinct in humans. Indeed, it should be noted that these analyses are skewed by the large amount of data derived from a single study in human sperm and blood (Jeffreys et al., 2013). Interestingly, we found very few instances where two *C*-type alleles could create an *A*-type allele suggesting that either very rare events or other mechanisms (such as mutation) are required to generate *A*-type from *C*-type alleles. Curiously, we found many cases where two *A*-type alleles could form a *C*-type allele; however, these required an average of nine template switches (compared to just two when both parental alleles were *C*-type; Figure 4C). Since most variants are a similar size to the parental allele (Supplementary Figure S4E), nine-switch events are likely to be very rare. Nonetheless, one variant in sperm did require nine switches (*Av:0540*, Man16S; Supplementary Figure S9). Together, it appears that *C*-type variants rarely arise in *A*-homozygotes and vice versa.

### Minor Variations at the *PRDM9* Binding Site Can Alter the Recombination Landscape in Humans

**FIGURE 5:**
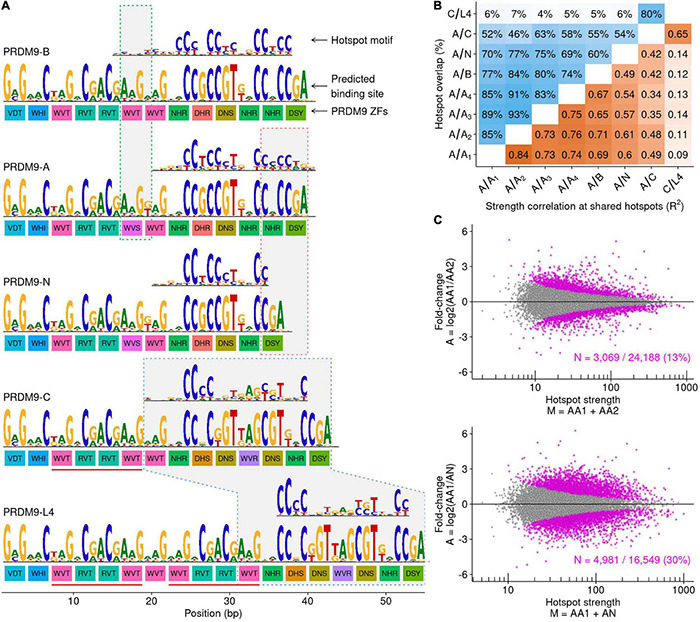
The *A*-variant *PRDM9-N* allele substantially perturbs the DSB landscape. **(A)** Schematic of the *PRDM9* ZF arrays and DNA binding preferences for each allele. For each allele, there are three rows of data. The colored boxes represent the amino acid sequences at the primary DNA contact residues for each C_2_H_2_ ZF (−1, 3, and 6 positions). These amino acids confer DNA binding specificity, and ZFs with different DNA contact residues are colored differently. The binding preference for each allele is predicted from the amino acid sequence using a polynomial SVM model (Persikov and Singh, 2014). Note that despite these predictions, not all ZFs are thought to contribute to *PRDM9* DNA binding. The best-scoring sequence motif identified at hotspots putatively defined by each allele is shown above each prediction. The green box highlights the region that differs between the *PRDM9-A* and *PRDM9-B* alleles. A single base pair change modifies one amino acid in the ZF array (Baudat et al., 2010). This slightly alters the predicted *PRDM9* binding. The red box outlines the region that differs between the *PRDM9-A* and *PRDM9-N* alleles. The *PRDM9-N* allele differs by one less ZF than the *PRDM9-A* allele. The blue box outlines the region that binds DNA in both the *PRDM9-C* and *PRDM9-L4* alleles. The four *PRDM9-C* ZFs underlined in red are duplicated in the *PRDM9-L4* allele. **(B)** Hotspots were identified in all samples, and overlapping hotspots were counted. The maximum reciprocal overlap is shown (top left; blue shading). At shared autosomal hotspots, the correlation between hotspot strength was calculated (bottom right; orange shading; *R*^2^ = squared Pearson correlation coefficient of log-transformed strength values). **(C)** MA plots depicting significantly different shared hotspot usage between (top) two *A/A* individuals and (bottom) the *A/A_1_* and *A/N* individuals. Each point represents one shared hotspot. Hotspots with differing strength are highlighted in magenta (Bonferroni-corrected binomial *P*-value < 0.001).

We next examined whether intra-type variation can drive substantial differences in the recombination landscape. Previous studies demonstrated that the *A*-type variant *PRDM9-B* (*PRDM9-B* differs from *PRDM9-A* by a single amino acid; Figure 5A; Baudat et al., 2010) had little impact on the recombination landscape in humans (Pratto et al., 2014b). In contrast, in C3H mice, the addition of a single ZF to the *Prdm9-B6* allele profoundly altered recombination localization, despite this ZF addition having little predicted impact on DNA binding (Smagulova et al., 2016). To further assess the impact of *PRDM9* variants on the patterns of meiotic recombination in humans, we generated and examined meiotic DSB maps for different variants within the *A*-type and the *C*-type *PRDM9* clusters.

Differences in the recombination landscape in individual men can be assessed by mapping meiotic DSB hotspots genome-wide. Hotspot locations are identified using a variant of ChIP-Seq to capture and sequence DNA bound by the DMC1 recombinase (DMC1 binds to single-stranded DNA at meiotic DSB hotspots; Khil et al., 2012). We previously mapped DSB hotspots in two *PRDM9-A* homozygous men (*A/A_1_*, *A/A_2_*), one *PRDM9-A/B* heterozygote (*A/B*), one *PRDM9-A/C* heterozygote (*A/C*; Pratto et al., 2014b), and recently in a *PRDM9-C/L4* heterozygous man (*C/L4*; Pratto et al., 2021). Here, we generated DSB maps in two further *PRDM9-A* homozygous men (*A/A_3_*, *A/A_4_*) and in a man heterozygous for the *PRDM9-A* allele and for an *A*-type variant (henceforth *PRDM9-N*; *Av:s:0053:M1S:A-A*; individual *A/N*). We compared these DSB maps to assess how the *A*-variant *PRDM9-N* allele and the *C*-type variant *PRDM9-L4* allele impact the meiotic recombination landscape.

The *PRDM9-N* allele was previously identified in the sperm of a *PRDM9-A/A* man as an *A*-derived variant, which can arise from a single templating switch from the *PRDM9-A* allele (Supplementary Figure S8B; Jeffreys et al., 2013). The differences between *PRDM9-A* and *PRDM9-N* reside in the C-terminus of the DNA binding site for *PRDM9-A*; *PRDM9-N* has one less ZF compared to *PRDM9-A* (Figure 5A and Supplementary Figure S8B). Thus, *PRDM9-N* likely binds a truncated version of the *PRDM9-A* sequence recognition motif (Figure 5A). Indeed, a truncated *PRDM9-A* consensus motif was identified from putative *PRDM9-N*-defined hotspots (DSB hotspots in the *PRDM9-A/N* individual that were not found in DSB maps from any of the *PRDM9-A/A* men; Figure 5B). The proportion of hotspots found in *A/N* but not in *A/A* individuals (23–31%; Figure 5B—top) exceeds the number of individual-specific hotspots in comparisons among *PRDM9-A/A* individuals (7–17%; Figure 5B—top and Supplementary Figure S10) and in comparisons between *PRDM9-A/A* and *PRDM9-A/B* individuals (16–26%; Figure 5B—top and Supplementary Figure S10). Thus, it appears that the binding preferences of *PRDM9-A* and *PRDM9-N* are substantially different and therefore define a small subset of N-specific hotspots. In addition to defining new hotspots, the presence of one copy of *PRDM9-N* substantially perturbs hotspot strength at *PRDM9-A*-defined DSB hotspots (Figures 5B—bottom, 5C and Supplementary Figures S10A,C). Together, these data demonstrate that the *A*-type *N* allele substantially alters the recombination landscape compared to the *A* allele. Thus, a single change in the *PRDM9* ZF array predicted to change DNA binding specificity and derived from a template switching event can strongly alter recombination patterns in humans.

A double template switch can give rise to the *L4* allele *via* the duplication of four ZFs of *PRDM9-C* (Figure 5A, red lines under *C* and *L4* alleles and Supplementary Figure S8C). However, in contrast to the previous example (*PRDM9-N* vs. *PRDM9-A*), this results in changes outside of the ZFs predicted to confer DNA-binding specificity (Figure 5A). Thus, the *PRDM9-C* binding site is retained fully in *PRDM9-L4*, and these alleles may bind to similar genomic targets. Consistent with this, we found a *PRDM9-C*-like motif at putative *L4*-defined hotspots (*C/L4* hotspots that were not found in the *A/C* individual; Figures 5A,B and Supplementary Figure S10). No additional motifs were found, implying that the addition of four ZFs had no detectable effect on DSB targeting. The 80% of hotspots shared between *C/L4* and *A/C* likely represent *PRDM9-C*-defined hotspots. Hotspot strength is well correlated at these shared hotspots, although below the correlation seen among *A/A* men (Figure 5B—bottom and Supplementary Figure S10). The slight perturbation of hotspot strength is likely caused by *PRDM9* heterozygosity in one or both individuals (Pratto et al., 2014b).

## Discussion

In humans, the hypervariable *PRDM9* gene determines the patterning of meiotic recombination. Understanding the patterning of recombination is key to inferring population structure and inferences made in genome-wide association studies. The DNA binding specificity of PRDM9 is encoded by a highly repetitive 84-bp minisatellite sequence array, and as a result, the *PRDM9* genotype cannot be inferred accurately from short-read sequencing. *PRDM9* genotyping still requires labor-intensive and low-throughput methods such as Sanger sequencing. As a result, our knowledge of *PRDM9* diversity has not greatly expanded since the advent of high-throughput sequencing, and thus, the population diversity of *PRDM9* in humans remains poorly understood.

In this work, we developed a novel strategy to efficiently genotype the *PRDM9* locus in hundreds of individuals using multiplexed long-read sequencing and have used this method to develop an extensive catalog of human *PRDM9* variation across seven populations. Our method substantially improves on previous methods to genotype *PRDM9* (Berg et al., 2010; Schwartz et al., 2014) by circumventing the need for labor-intensive gel extraction, amplicon isolation, and Sanger sequencing as well as by increasing the throughput *via* barcoded sample multiplexing of a large pool of amplicons. Labor-intensive amplicon isolation is required for Sanger sequencing-based approaches to genotype *PRDM9* because the repetitive nature of *PRDM9* causes PCR amplification artifacts. We perform this clean-up *in silico* instead, by retaining only reads that span the entire ZF array. Although this is effective for the vast majority of samples, PCR artifacts are still a source of error using our strategy. An initial concern of using long-read sequencing for *PRDM9* genotyping was that the error rate may be prohibitively high to accurately phase *PRDM9* alleles that differ by as little as a single nucleotide. However, we found that with sufficient depth of coverage, this was a minor concern. Finally, we compared the accuracy of the two major long-read sequencing platforms (PacBio and Oxford Nanopore) for genotyping *PRDM9*. We found comparable accuracy using both methods and suggest that both platforms are sufficiently accurate for *PRDM9* genotyping. Other aspects of these platforms such as cost and accessibility are likely more important considerations than the accuracy of sequencing.

Utilizing our new methodology, we inferred the *PRDM9* diploid genotypes of 720 individuals from seven human populations spanning four continents: Africa (LWK and YRI), Asia (CHB and PJL), Europe (FIN and TSI), and South America (PEL). This greatly expands on previous *PRDM9* surveys in several ways; first, this is by far the largest survey of human *PRDM9*; second, unlike previous surveys, we analyzed the diploid *PRDM9* genotype; and third, in contrast to previous studies that had a European population bias (Baudat et al., 2010; Berg et al., 2010; Parvanov et al., 2010), we captured a large swath of human genetic diversity. We identified 69 distinct *PRDM9* alleles including 32 novel alleles. We also identified 13 alleles that were previously only seen as *PRDM9* variants in sperm or blood (Jeffreys et al., 2013). This implies that the hundreds of *PRDM9* alleles previously discovered only in human sperm/blood represent a font of human *PRDM9* diversity.

Consistent with previous studies, we found that *PRDM9-A* was the predominant allele in all populations and that *PRDM9* diversity was exceptionally high in African populations (Berg et al., 2011; Hinch et al., 2011). The other major *PRDM9* allele, *PRDM9-C*, was previously thought to be found mostly in Africa. Instead, our study reveals that *PRDM9-C* is present in many populations but depleted in European populations. Unlike *PRDM9-C*, *C-type* alleles are found almost exclusively in Africa. This may suggest that *PRDM9-C* was present in individuals that emerged from the human migration bottlenecks that have likely constrained *PRDM9* diversity in non-African populations. Unique to our study, we also found that some alleles of *PRDM9* appear to be segregated by population. For example, *PRDM9-B* is notably enriched in the Han Chinese (CHB) population. This increased prevalence may reflect some advantage to having this allele; however, *PRDM9-B* only differs from *PRDM9-A* by a single nucleotide, which has little impact on meiotic DSB patterning (Pratto et al., 2014a; Altemose et al., 2017). Alternatively, differences may simply reflect genetic drift. The most intriguing population-specific allele is *PRDM9-D*, which was confined to the Finnish population in our study. *PRDM9-D* was previously shown to coincide with hyper-variation at the *PRDM9* ZF array (Jeffreys et al., 2013); however, we did not see elevated *PRDM9* diversity in the FIN population. Thus, if *PRDM9-D* is causing hyper-variation of *PRDM9*, it has not manifested in the population at the levels assessed here. This could also simply reflect the rarity of this allele as it was found in just six individuals. As was seen previously, numerous rare alleles were found in the two African populations, LWK and YRI, and not the others. Differences between the two African populations were also seen, such as *PRDM9-L14* enrichment in LWK and *PRDM9-L19* enrichment in YRI. Importantly, given the large number of low-frequency alleles in both African populations, a deeper study of more individuals is required to assess the true extent of differences between these populations. Furthermore, both African populations studied are related to Bantu-speaking peoples. Thus, we are likely still substantially underestimating the diversity of *PRDM9* alleles in Africa.

Although our strategy makes *PRDM9* genotyping more tractable at scale, the ability to infer the *PRDM9* genotype from nearby SNPs would allow rapid genotyping of this locus. In several previous studies, SNPs were found to be associated with variation in the recombination landscape (Kong et al., 2010; Hinch et al., 2011), and we expanded upon these studies by demonstrating that each of the four major *PRDM9* alleles are strongly associated with SNPs in the surrounding region. A caveat of these findings is that since *PRDM9* variation arises from template switching at the ZF array, new alleles can arise on the same haplotype background as another allele. Indeed, the SNPs associated with *PRDM9-A* are also associated with other *A-type* (but not *C-type*) alleles. Thus, depending on the frequency of the allele, and on the number of variants derived from that allele, the utility of SNP-based imputation will vary.

*PRDM9* variants can be found both somatically (blood) and in the germline (sperm) (Jeffreys et al., 2013), and variant alleles are often defined by ZF gains or losses. Consistent with a previous work, we could explain all *PRDM9* diversity in men with a known *PRDM9* genotype by allowing for template switching between the two *PRDM9* alleles. Using this algorithm, most of the *PRDM9* alleles in the studied human populations could be derived from template switching between others. However, we found that creating an *A-type* allele from two *C-type* alleles or a *C-type* allele from two *A-type* alleles is very unlikely to occur. This mechanism would seem to reinforce the broad *A*-type/*C*-type clusters of human *PRDM9* alleles that are seen in our study. Thus, it seems possible that all the human *PRDM9* alleles found to date represent the mutational drift of two alleles in the population. Single-nucleotide polymorphisms add another layer of complexity to the relatedness of *PRDM9* alleles. The formation of novel alleles by SNPs was not modeled in our work but has the potential to dramatically alter the binding preference of a *PRDM9* allele. Thus, SNPs may be the key to generating truly new *PRDM9* variants.

The exact mechanism(s) by which *PRDM9* diversity arises remain unknown (Jeffreys et al., 2013). A common mechanism, such as error-prone DNA replication, may give rise to this variation in somatic and germ cells; however, many *PRDM9* variants in sperm require inter-allelic exchanges. Our data imply that inter-allelic template switches are a major source of *PRDM9* variation and that inter-allelic template switches alone can explain almost all observed variants in sperm. The spatial alignment of homologs would be required to allow for inter-allelic interactions during DNA replication, and interestingly, the parental homologs partially align in meiotic S-phase (at least in mice; Boateng et al., 2013). Furthermore, alignment is most pronounced in sub-telomeric DNA, and *PRDM9* resides on the distal p-arm of chromosome 5 in humans. It is also possible that new alleles arise as the result of gene conversion during recombination. None of the alleles studied to date appear to create a DSB hotspot sufficiently close to the ZF array to allow canonical inter-homolog interactions during recombination (closest hotspot is ∼5 Kb away in the *A/C* individual and tens of kilobytes in *A/A* individuals). However, since the formation of a *PRDM9* variant is a rare event, non-canonical interactions or weak hotspots below the detection threshold of current methods could be responsible. Men carrying *PRDM9-C, C*-type alleles, or *PRDM9-D* have an elevated rate of *PRDM9* variant formation in sperm (Jeffreys et al., 2013), and this could occur if these alleles occasionally initiate recombination near the ZF array. Alternatively, the elevated variant formation in these men may stem from other differences in populations enriched for these alleles. One final (and speculative) hybrid hypothesis is that replicative errors in meiosis can be repaired *via* a mechanism that involves the homolog, thus creating more frequent and more diverse variants than in somatic cells.

*PRDM9* localizes meiotic DSBs and recombination in human genomes. As a consequence, the binding sites of *PRDM9* are rapidly destroyed by gene conversion during DNA repair (for review, see Grey et al., 2018). This process, known as hotspot erosion, will purge strong *PRDM9* binding sites from the genome and, thus, may favor the emergence of new variants of *PRDM9* with different DNA binding specificity (Myers et al., 2010; Lesecque et al., 2014). Whether intra-type variation (*A*-type/*C*-type alleles) can sufficiently diversify *PRDM9* binding sites to confer this benefit is unknown. The *PRDM9-B* allele differs from *PRDM9-A* by a single amino acid outside the DNA binding site (Baudat et al., 2010; Jeffreys et al., 2013), but this change has little impact on DSB hotspot localization (Pratto et al., 2014b). In contrast, the *PRDM9-N* allele (*Av:s:0053:M1S:A-A*), which differs from *PRDM9-A* at the C-terminus of the *PRDM9-A* binding site, perturbs the DSB hotspot landscape and defines a new subset of what appears to be *N*-defined hotspots. These observations suggest that *PRDM9-N* has a slightly different binding preference to *PRDM9-A*. Alternatively, we cannot exclude that *PRDM9* heterozygosity is responsible for these perturbations, as heterozygosity *per-se* can affect hotspot usage to a similar degree (Pratto et al., 2014b) and the N-defined hotspots were only mapped in an *A/N* heterozygous man. Finally, the *C-type PRDM9-L4* allele not only has four ZFs more than *PRDM9-C* but also retains the intact *PRDM9-C* binding site. Almost all DSB hotspots in a *C/L4* heterozygous man were also seen in an *A/C* heterozygote, suggesting that despite the substantial length difference between their ZF arrays, *PRDM9-C* and *PRDM9-L4* define similar hotspots. Thus, from these samples, only the variant that changes the documented *PRDM9* binding site can alter DSB hotspot targeting. Nonetheless, in mice, it has been shown that the removal of ZFs with low binding specificity can still greatly impact *PRDM9* binding (Smagulova et al., 2016). Together, these data suggest that even relatively minor changes to the *PRDM9* ZF array may impact the recombination landscape. *PRDM9* binding remains poorly understood (Billings et al., 2013), but given the diversity of alleles found in many human populations, far more work is required to understand how small changes in the DNA binding specificity could impact the DSB landscape.

In summary, the methodology we present in this study allowed for accurate and high-throughput sequencing of the highly repetitive and difficult-to-genotype *PRDM9* locus. This strategy may also be adapted to study other minisatellite or repetitive loci in the genome. These data offer a glimpse at the previously under-appreciated diversity of *PRDM9* in a sampling of human populations and open the door to far more detailed studies of this and other minisatellite loci in the future.

## Materials and Methods

### Human Population Samples

The DNA samples were obtained from the NHGRI Sample Repository for Human Genetic Research at the Coriell Institute for Medical Research: repository numbers are presented in Supplementary File S1. In summary, we obtained genomic DNA from 811 individuals from seven human populations defined in the 1000 Genomes Project/HapMap Project (list below and Supplementary File S1; Coriell Institute; samples are deidentified). Genomic DNA was purified from either blood or immortalized lymphocytes/fibroblasts using either the Qiagen Autopure LS instrument or by a modified Miller’s salting out procedure (performed at the repository).

Population reference ID and nomenclature and the number of individuals genotyped are as follows:

MPG00013—Yoruba in Ibadan, Nigeria (YRI)

YRI Trios—Yoruba in Ibadan, Nigeria (YRI-trios)

MPG00008—Lyhya in Webuye, Kenya (LWK)

MPG00007—Toscani in Italia (TSI)

MPG00001—Finnish in Finland (FIN)

MPG00011—Peruvian in Lima, Peru (PEL)

MPG00017—Han Chinese in Beijing, China (CHB)

MGP00020—Punjabi in Lahore, Pakistan (PJL).

### Amplification of *PRDM9* C_2_H_2_ Zinc Finger Array

*PRDM9* ZF array sequences from all samples were amplified with primers from Berg et al. (2010). No known human SNPs occur within these primer sequences, and they are fully conserved in other mammalian species (mouse, dog, and elephant; single nucleotide change in each primer in macaque). The primer sequences used are as follows:

Forward: 5′-TGAGGTTACCTAGTCTGGCA-3′(hg38 5:2352 5987-23526006)

Reverse: 5′-ATAAGGGGTCAGCAGACTTC-3′(hg38 5:2352 7867-23527886).

LongAmp Taq 2X Master Mix (M0287) from New England Biolabs Inc. was used for PCR amplification. Post-amplification, samples were individually tested for successful amplification and low presence of polymerase slippage (presence of DNA laddering/smearing) by running on agarose gel electrophoresis (Supplementary Figure S1). Samples were re-amplified if there was extensive DNA laddering/smearing by visualization. Based on the Genome Reference Consortium Human Build 38 (*PRDM9* allele with 13 ZFs), the final amplified product was 1,899 bp, which contained the 1,092-bp C_2_H_2_ ZF array, 670 bp of upstream flanking sequence, and 137 bp of downstream flanking sequence to the *PRDM9* ZF array. Of note, the total length of the final amplified product varied based on the number of ZFs present in the *PRDM9* allele. Samples were then pooled and prepared for multiplexing.

### Multiplexing of *PRDM9*-Amplified Samples

We performed dual-barcoding in order to multiplex and sequence amplicons targeting the *PRDM9* ZF array. The first round of barcoding was done by adding unique DNA barcode sequences to the 5′-end of the primers detailed above, totaling eight primer pairs:

Barcode 1: 5′-ATCACGATCACG-3′

Barcode 2: 5′-CGATGTCGATGT-3′

Barcode 3: 5′-GATCAGGATCAG-3′

Barcode 4: 5′-CTTGTACTTGTA-3′

Barcode 5: 5′-ACAGTGACAGTG-3′

Barcode 6: 5′-GCCAATGCCAAT-3′

Barcode 7: 5′-CAGATCCAGATC-3′

Barcode 8: 5′-ACTTGAACTTGA-3′.

After amplification and the addition of the first barcode, samples were pooled in groups of eight (each sample tagged with a separate barcode sequence) and subjected to a second round of barcoding. The second round of multiplexing was performed using the PCR Barcoding Expansion 1-96 kit (EXP-PBC096) from Oxford Nanopore Technologies (ONT), Inc. following the protocol detailed on their website^1^ [PCR barcoding (96) amplicons]. In short, adapter sequences are ligated to amplicons and are used as the priming sequence for a second round of PCR amplification that adds one of 96 commercially available barcode sequences.

This barcoding scheme allows for multiplexing of 768 samples at one time, 8 primer-barcodes × 96 ONT PCR barcodes. Post-multiplexing, all samples are pooled and prepared for long-read sequencing.

### Nanopore Sequencing

Sequencing libraries were prepared using the ligation sequencing kit (1D; SQK-LSK109) or 1D^2^ sequencing kit (SQK-LSK309) from Oxford Nanopore Technologies. Library preparation was performed as detailed by the protocols on ONT’s website (see footnote 1). All nanopore sequencing experiments were run on a MinION sequencer with R9.5.1 (1D^2^: FLO-MIN107) or R9.4.1 (1D: FLO-MIN106) flow cells.

### PacBio Sequencing

Pooled samples were prepared using the SMRTbell Express Template Prep Kit 2.0 (Pacific Biosciences, CA, United States) and sequenced using the PacBio Sequel II System to generate CCS PacBio reads. Sequencing was performed with a 0.5-h pre-extension and 10-h recording time, and a second sequencing run was performed with a 2-h pre-extension and 30-h recording time.

### Basecalling and Demultiplexing of PacBio Circular Consensus Sequencing Reads

Basecalling was performed using the PacBio CCS tool (bioconda channel pbccs-4.2.0.0) and default parameters. The sequences of all possible barcode combinations from our dual barcoding approach were appended to a barcodes FASTA file. The reverse complement of each barcode was also included. Demultiplexing was performed using the PacBio lima tool (bioconda channel pblima-1.11.0) and the following command line arguments: –ccs –guess 45 –peek 10000 –guess-min-count 5 –different –score-full-pass. Only reads flanked by a barcode on one side and its reverse complement on the other were retained.

### Base Calling and Demultiplexing of Oxford Nanopore Reads

To identify sequencing reads derived from each individual, we performed read demultiplexing using Guppy v3.1.5. This first involved base calling (with standard parameters), followed by two rounds of demultiplexing to identify the outer and inner barcodes. The first round of demultiplexing identified the outer barcode as follows:

guppy_barcoder –compress_fastq -i {guppy output}

-s demux

–arrangements_files barcode_arrs_pcr96.cfg

–min_score 50 –front_window_size 300

–rear_window_size 300

–trim_barcodes

The second round of demultiplexing was then performed on each of the files generated from the first round:

guppy_barcoder –compress_fastq -i {round 1 barcoding FAST5}

–arrangements_files custom_12bp.cfg

–min_score 70 –front_window_size 100

–rear_window_size 100

–trim_barcodes

We used the Oxford Nanopore development basecaller Bonito (v.0.2.3) for base calling as it is more accurate than Guppy, the production basecaller (Silvestre-Ryan and Holmes, 2020). Specifically, we found that the Guppy base calling accuracy for CpG dinucleotides in particular contexts was insufficient to confidently infer *PRDM9* genotypes using our methods (not shown). Reads from each individual were grouped and base called separately using Bonito (v.0.2.3) and default parameters.

### *PRDM9* Genotyping From Long Reads

Genotyping *PRDM9* from long reads presents two challenges: first, PCR artifacts of the wrong length should be purged and second, alleles that differ by a single base pair should be identifiable. We therefore devised a strategy to identify all reads with an intact ZF array and then to use multiple sequence alignment to call variants. Note that a preliminary study using the Guppy basecaller could not be used for this approach because of systematic base calling errors at CpG dinucleotides in a particular context.

The *PRDM9* ZF array was first identified for each sequencing read. The sequences immediately flanking the ZF array were identified using a Smith–Waterman local alignment tool (Water, EMBOSS suite; Rice et al., 2000). The flanking sequences are as follows:

*PRDM9* zinc finger array 5′ flanking sequence:

CACAGCCGTAATGACAAAACCAAAGGTCAAGAGATCA AAGAAAGGTCCAAACTCTTGAATAAAAGGACATGGCAGA GGGAGATTTCAAGGGCCTTTTCTAGCCCACCCAAAGGAC AAATGGGGAGCTGTAGAGTGGGAAAAAGAATAATGGAA GAAGAGTCCAGAACAGGCCAGAAAGTGAATCCAGGGAA CACAGGCAAATTATTTGTGGGGGTAGGAATCTCAAGAAT TGCAAAAGTCAAGTATGGAGAG.

*PRDM9* zinc finger array 3′ flanking sequence:

GATGAGTAAGTCATTAGTAATAAAACCTCATCTCAATA GCCACAAAAAGACAAATGTGGTCACCACACACTTGCACA CCCCAGCTGTGAGGTGGCTTCAGCGGAAGTCTGCTGAC CCCTTATATTCCCCGAGAGTATAAAGAGATCGGAAATAAC TGATTAAACAAATCCGCCACTTTCATGACTAGAGATGAG GAAGAACAAGGGATAGTTCTGTAAGTGTTCGGGGGACAT CAGCATGTGTGGTTCTTTC.

These flanking sequences were used to define the start and end points of the *PRDM9* ZF array. Sequences lacking either flanking sequence were discarded. BLAST (Altschul et al., 1990) (bioconda channel—blast-2.10.1) was subsequently used to identify the position of C_2_H_2_ ZFs within each sequencing read containing a full-length array. For the BLAST search, we used the set of all published *PRDM9* ZFs (see below) as a search query. BLAST used the following command line arguments: blastn -word_size 7 -max_hsps 200 -num_alignments 20000 -evalue 1 -culling_limit 20000. Partial hits to ZFs were sometimes obtained because of gaps in long reads. These hits were padded to 84 nucleotides with Ns. Only reads with a contiguous array of C_2_H_2_ ZFs, flanked immediately by the expected 5′ and 3′ sequences, were retained. Individuals with <100 × coverage were not processed further. The size of the ZF arrays were inferred as follows:

**Table:** 

**#**	**Rule**	**Length (hap 1)**	**Length (hap 2)**
1	All zinc finger arrays had *i* zinc fingers (*f*_*i*_ = 1)	*i*	*i*
2	*f*_*i*_ + *f*_*j*_ ≥ 0.7 AND *f*_*i*_ / *f*_*j*_ < 2	*I*	*j*
3	*f*_*i*_ + *f*_*j*_ ≥ 0.7 AND *f*_*i*_ / *f*_*j*_ < 3 AND *f*_*j*_ / *f*_*k*_ > 2	*i*	*j*
4	*f*_*i*_ + *f*_*j*_ ≥ 0.7 AND *f*_*i*_ / *f*_*j*_ > 3	*i*	*i*
5	*f*_*i*_ ≥ 0.7	*i*	*i*
			

Where *f*_*i*_, *f*_*j*_, and *f*_*k*_ are the frequencies of the most frequent (*i*), second most frequent (*j*), and third most frequent (*k*) ZF arrays. Rules are processed consecutively; thus, an individual where the ZF array lengths can be inferred using rule 1 will not be tested by further rules.

ZF arrays matching the expected haplotype lengths were retained, and we attempted to infer both *PRDM9* haplotypes for each individual. Individuals where the two ZF arrays differed in length were straightforward, as the diploid genotype could be simply inferred from the consensus sequences of each ZF array. Sequences that had any nucleotide with a consensus sequence frequency (not including N’s or gaps) <0.6 were discarded. Individuals where both ZF arrays were the same length were processed as follows: the consensus sequence across the ZF array was determined and the consensus frequency (*f*_*c*_) for each nucleotide position was calculated (consensus nucleotide/total sequences; N’s and gaps were excluded from the totals). Any ZF array with ≥ 1 nucleotide having *f*_*c*_ < 0.7 was considered potentially heterozygous. To test for heterozygosity, each ZF array sequence was reduced to only the sequence at the heterozygous loci. A pairwise distance matrix was constructed between all pairs of sequences (distance = # mismatches) and was used for hierarchical clustering (R hclust function). The optimal number of clusters (*n*) was determined as the number of clusters that gave the minimum within-cluster mean distance (tested; 1 ≤ *n* < 20). Sequences in the largest two clusters likely represent the two major haplotypes, while sequences in other clusters (if *n* > 2) likely represent sequences with sequencing errors. Finally, we tested the internal consistency of each haplotype cluster as we did initially for all sequences; if either putative cluster yielded any nucleotide with *f*_*c*_ < 0.7, then we conclude that the genotype could not be inferred for that allele.

### Nomenclature of New *PRDM9* Alleles and Zinc Fingers

*PRDM9* alleles that did not match any of the previously published human *PRDM9* alleles were designated a name of “M#,” where # represents a simple numerical index (Supplementary File S3). New ZF sequences were named “!%,” where % represents an uppercase letter (Supplementary File S2).

### Obtaining Published *PRDM9* Alleles and Zinc Finger Sequences

We obtained the DNA sequences for all human *PRDM9* alleles (in Supplementary File S3) and C_2_H_2_ ZF sequences from Baudat et al. (2010), Berg et al. (2010), and Jeffreys et al. (2013) (details in Supplementary Files S2, S3). Most of the documented *PRDM9* alleles were derived from the supplementary information of a study of *PRDM9* variants in human sperm (Jeffreys et al., 2013). These unnamed variants were assigned a five-part name as follows:

(1) **Variant type:**
  The parental *PRDM9* allele from which this variant was likely derived. Recombinant variants and variants of unknown origin are designated Rv and Uv, respectively.
(2) **(s)imple or (c)omplex:**
  Simple events can be explained by a single event, complex cannot.
(3) **Allele number:**
  A unique numeric index for each allele.
(4) **Man ID (S)perm or (B)lood:**
  Identifier for the tissue donor as well as the origin material type.
(5) **Parental *PRDM9* genotype:**
  First allele-second allele (i.e., *A-L20*).

For example,

*Av:c:0065:M2S:A-L20* = *A*-variant : complex : #65 : Man 2 Sperm : *A / L20* genotype.

Allele names have been shortened to variant type:allele number in figures due to space constraints, e.g., in figures, *Av:c:0065:M2S:A-L20* = *Av:0065*.

For the other *PRDM9* variants, the name from the previous study was retained. The C_2_H_2_ ZFs of *PRDM9* were named using a single-character code; however, to allow for the expansion of the ZF repertoire in this study, we re-named each ZF using a two-character code (Supplementary File S2).

### Identification of Single-Nucleotide Polymorphisms Associated With *PRDM9* Alleles

For this study, we used data from individuals who had a diploid inferred *PRDM9* genotype and for whom hg38 SNP data were available in the 1000 Genomes Project VCF files (1000 Genomes Project Consortium et al., 2015; 27022019 release). The 59 YRI “children,” with parents among the other YRI individuals, were excluded. This yielded data from 649 individuals.

We examined SNPs within ±20 Mb of the *PRDM9* transcript start and excluded INDELs, SNPs with a minor allele frequency (MAF) < 2%, SNPs within the coding region of the *PRDM9* ZF array, and SNP loci with missing information. The resultant dataset contained 151,944 SNPs. A similar experiment using all of chr5 yielded analogous results (not shown). To perform phenotype–genotype association analyses, allowing for population stratification, we used PLINK (v1.07) (Purcell et al., 2007) with the following command line arguments: –assoc –all-pheno –allow-no-sex –mh –within populations.txt. We defined phenotypes as individuals with at least one copy of a given *PRDM9* allele. Thus, heterozygotes and homozygotes were treated equally. Multiple associated SNPs were identified for each phenotype. For analyses, the SNP with the lowest *P*-value was used. In cases where multiple SNPs had the same *P*-value, rs6889665 was chosen if it was among the top-scoring SNPs; otherwise, one SNP was chosen at random. This random choice did not affect downstream analyses. The three SNPs with the highest association score for *PRDM9-C* (rs77023486, rs141586808, and rs138354146) did not show subsequent enrichment among *PRDM9-C* carriers. Association estimates are sensitive to rare SNP variants, and since all three SNPs had MAF ≈3.5%, they were excluded from downstream analyses. All SNPs associated with each phenotype are given in Supplementary File S4.

### Validating Novel *PRDM9* Alleles Using Published Exome Sequencing Data

We obtained exome sequencing data from the 1000 Genomes Project (Google Cloud mirror^2^) for individuals carrying at least one novel allele identified in this study. We used samtools view (v.1.12) to extract only the reads that aligned to the terminal exon of *PRDM9*, which contains the ZF array (locus extracted from hg38: chr5:23525000-235320000). We then created a FASTQ from these reads using bedtools bamtofastq (v2.30.0). Using minimap2 (v2.20; arguments –x sr –a), reads were aligned to a FASTA file containing one entry for each distinct *PRDM9* ZF found in this study. Reads were then filtered by the CIGAR string to remove reads with mismatches or with <73 bp aligned.

### *In silico* Analysis of *PRDM9* Allele Formation

Previously, the evolutionary relatedness of *PRDM9* was inferred using classical sequence alignment with either no modifications (Kono et al., 2014) or using modifications that included penalties for amplifications and contractions of the minisatellite-like *PRDM9* ZF array (Bérard et al., 2007; Bonhomme et al., 2007; Buard et al., 2014). Unmodified sequence alignment is not suited to assessing the relatedness of *PRDM9* alleles, but it is equally unclear if the added complexity of the latter method serves as an accurate model for *PRMD9* relatedness as this strategy limits amplifications/contractions to a single ZF and does not allow for new ZF variants that arise from splicing between ZF midpoints. Instead, we devised a simpler approach that assumes that template switching is the major means by which new *PRDM9* ZFs arise (Supplementary Figure S8). We made no inferences about the underlying mechanism, as template switching may result from a combination of replicative errors, gene conversion, and/or recombination. We compare the DNA sequence of a *PRDM9* ZF array (child) to the DNA sequence of the putative parental alleles (parents) to know if the child allele can arise from template switching between the parental alleles. Algorithmically, this is achieved as follows (see also Supplementary Figure S8):

(1) Find the longest match between the 5′ of the child and the 5′ end of parent 1.
(2) Truncate the child allele by removing the matched region.
(3) Find the longest common subsequence between the 5′ end of the truncated child allele and any location within the parent 2 allele (the match does not have to be at the 5′ end).
(4) Repeat 2–3, alternating between parental alleles until the truncated query matches the 3′ sequence of the active parental allele.
(5) Repeat 1–4, starting from the second parental allele.

*NOTES:*

(I) All matches are required to be longer than 15 nt.
(II) This approach allows for unlimited template switches; however, it is not clear if such multi-switches are biologically feasible.

The script for allele formation is available at https://github.com/kevbrick/prdm9_TS.git.

### DMC1-SSDS

Testicular samples were obtained from a commercial source (Folio Biosciences, Ohio). From a biopsy, 0.3 mg of normal adjacent tissue was obtained. Genomic DNA was extracted from the testicular samples before fixation with the DNeasy Blood and Tissue Kit (Qiagen). *PRDM9* genotype was obtained by amplifying, cloning, and Sanger sequencing the ZF array as described in Pratto et al. (2014b). The rest of the sample was directly thawed in 1% paraformaldehyde and gently dissociated. DMC1-SSDS was performed as described in Pratto et al. (2014b) and Brick et al. (2018). The discontinued anti-DMC1 antibody (Santa Cruz, cat#sc 8973) was used for this experiment.

Paired-end Illumina sequencing reads were aligned to the human reference genome (hg38) using a variant of the ssDNA alignment pipeline developed in Khil et al. (2012). First- and second-end reads were independently aligned to the genome using BWA-MEM 0.7.12 (Li, 2013). The captured fragment for each read pair was inferred, and the 5′ end of the two reads were compared to detect ssDNA stem-loop structures that were generated during library construction (Khil et al., 2012). Unambiguous ssDNA-derived reads were defined as previously described (Khil et al., 2012; Brick et al., 2018) and were retained for further analyses. Reads that were not unambiguously derived from ssDNA were discarded. DSB hotspots were identified from anti-DMC1 SSDS experiments using MACS (Zhang et al., 2008) version 2.0.10 and matched control data. The following MACS arguments were used: –nomodel; –shiftsize: 400; –bw: 1000; –q: 0.1. The peak sets obtained were then filtered to remove peaks that occurred on unassembled contigs and peaks that overlapped centromeres or centromeric repeats.

The scripts and analytic pipeline used for data analysis are available on Zenodo at DOI: 10.5281/zenodo.5149066.

## Data Availability Statement

Sequencing data from this study are deposited at the Gene Expression Omnibus (GEO) under accession number GSE166483 (https://www.ncbi.nlm.nih.gov/geo/query/acc.cgi?acc=GSE166483).

## Ethics Statement

Ethical review and approval was not required for the study on human participants in accordance with the local legislation and institutional requirements. Written informed consent for participation was not required for this study in accordance with the national legislation and the institutional requirements.

## Author Contributions

BA, KB, FP, MH, and RC-O contributed to design and conception of the study. BA performed the multiplexing and long-read sequencing of human samples. FP performed the ChIP-seq (SSDS) experiments from human testes samples. KB implemented the long read sequencing pipeline and data visualization. BA and KB wrote the manuscript. RC-O revised the manuscript and approved the final version. All the authors reviewed the final version of the manuscript.

## Conflict of Interest

The authors declare that the research was conducted in the absence of any commercial or financial relationships that could be construed as a potential conflict of interest.

## Publisher’s Note

All claims expressed in this article are solely those of the authors and do not necessarily represent those of their affiliated organizations, or those of the publisher, the editors and the reviewers. Any product that may be evaluated in this article, or claim that may be made by its manufacturer, is not guaranteed or endorsed by the publisher.

## Footnotes

1
https://nanoporetech.com

2
https://genomics-public-data/ftp-trace.ncbi.nih.gov/1000genomes/ftp/

## Supplementary Material

Supplementary Figure 1 PCR amplification biases are removed *in silico*. Agarose gels for post-PCR amplified *PRDM9* ZF arrays. Raw gel images are shown in **(A,C)**. Per-lane quantification is shown in **(B,D)**. Pixels used for quantification are shown beneath each histogram. Gel quantification was performed using the R imager package. For each sample/lane, the middle plot depicts the read length distribution. The lower plot depicts the inferred lengths of *PRDM9* ZF arrays from all sequencing reads used for final genotyping. For all individuals with sufficient coverage, we observe either one or two peaks in *PRDM9* ZF array length. *PRDM9* ZF arrays that do not coincide with the major peaks likely represent PCR artifacts that serendipitously created an erroneous, but complete, ZF array. These are a small minority of reads for all individuals. Coverage above 2,001 reads is not considered. **(A,B)** Twenty-four individuals with varying degrees of “laddering.” **(C,D)** Sixteen individuals with various amplification issues. Lane 2: cleanly amplified product. Lane 4: little/no amplification. Lane 12: smearing (unknown reason). DNA from lanes 4, 5, 9, and 12 were not used for sequencing. Further amplification experiments were performed and used for sequencing. The LCL gDNA control was DNA from an LCL cell line used as an amplification control. Lanes 6 and 8 contain DNA amplified from an *A/A* and *A/N* individual, respectively.

Supplementary Figure 2 The diploid *PRDM9* genotype can be inferred from long-read sequencing data for most individuals. **(A–C)** Inferred ZF array sizes (in # of ZFs) using PacBio or Nanopore reads. Each individual ID is shown in black. The inferred genotypes from Nanopore (blue) and PacBio (orange) are shown alongside the inferred diploid ZF sizes. Concordant alleles are replaced with hyphens (–). New alleles are not named, as the nomenclature is determined after pooled genotyping. **(A)** Six representative individuals with concordant genotypes. **(B)** Five individuals had discordant genotypes where the number of ZFs matched. Three out of five are the result of differences at a single nucleotide (NA18535, NA19019, and NA19030). For the other two individuals, the discordant alleles differ by 3 nt (NA20522; *A* vs. *L24* allele) and 21 nt (NA19437; *A* vs. *L12* allele). **(C)** Differing ZF lengths were the cause of the eight remaining discordant genotypes. This mostly occurs because of insufficient sequencing depth or under-representation of an allele with one technology (HG03640, NA08873, NA19026, and NA19473). In three cases, the amplicon size distribution differed between the reads from the two technologies (HG00311, NA19240, and NA19043). For the remaining individual (NA19470), it appears that a 12-ZF amplicon was erroneously considered a valid allele for PacBio. **(D)** Detection bias for shorter *PRDM9* alleles. For all heterozygous individuals, the percentage of reads for each genotype was calculated. These percentages are shown for alleles of different sizes. Absent any bias, all boxplots should have a median at 50%. The reduced coverage for longer alleles is seen for both technologies and therefore is most likely derived from the PCR step during library preparation. Thirteen-ZF alleles have apparently higher coverage; however, this is likely because most heterozygotes with one long allele also contain a 13-ZF allele (i.e., *PRDM9-A*). In these individuals, the relative amount of 13-ZF sequences will often be above 50%. **(E)** *PRDM9* inference from pooled sequencing reads. The diploid genotype is considered successfully inferred if both *PRDM9* alleles are confidently determined from sequencing data. Most individuals lacking a *PRDM9* genotype were among the 59 YRI individuals used for trio analyses (YRI-Trios) who were sequenced to shallower depth. **(F)** Failed samples have substantially fewer raw sequencing reads. Points depict each individual. Boxplots show the interquartile range with the median depicted as the solid dividing line. **(G)** Not all raw sequencing reads can be used for inferring genotypes. Failed samples have substantially fewer reads with a contiguous *PRDM9* array. In addition, “no genotype” individuals are absent from **(F,G)** if no reads were obtained (mostly due to failed barcoding for some samples).

Supplementary Figure 3 Most novel alleles are validated using exome sequencing data. We extracted exome sequencing reads that mapped to the *PRDM9* ZF array (1000 Genomes Project), then aligned these to all possible *PRDM9* ZFs (see section “Materials and Methods*”*) for each individual. Short-read exome sequencing data were available, and analyses are presented here for 48/53 individuals who have at least one previously unannotated *PRDM9* allele. Most exome sequencing was performed with 75-bp reads. Reads aligned with <72 bp or with mismatches were discarded. Since each ZF is 84-bp long, some reads may still map ambiguously despite these stringent criteria. We compared read density for ZFs found in both alleles (red), ZFs found uniquely in each allele (blue), and ZFs that were not found in either allele (green). The 95th and 98th percentile for ZFs not found in either allele are indicated with green dotted and dashed lines, respectively. Coverage is normalized for the number of each ZF present in an individual; coverage for ZFs not in the individual were normalized by 1. For three individuals, no reads passing our criteria were identified (NA19145, NA18527, and HG00359), and for five further individuals (HG00377, HG00312, NA20787, NA20768, and NA20769), very few reads were identified. For all remaining individuals, normalized read coverage for the novel allele(s) exceeded the 95th percentile of coverage for the control alleles.

Supplementary Figure 4 *PRDM9* alleles are categorized as *A*-type and *C*-type. **(A)** *PRDM9* alleles found in human populations broadly cluster into two groups defined by similarity to the PRDM9-A or PRDM9-C binding site. The binding site for each allele was predicted (Persikov and Singh, 2014), and alleles were clustered using the position weight matrix for each allele (motifStack function of R motifPiles library). The regions matching the known binding residues for PRDM9-A (purple) and PRDM9-C (green) are highlighted. Motifs were manually aligned to highlight these loci. **(B,C)** A distance metric that measures the similarity to the amino acid sequence that defines *PRDM9-A* and *PRDM9-C* binding (distance = # of mismatches + # of gaps from a BLAST alignment) was used. Alleles left of the diagonal line are alleles with binding sequences more similar to the *PRDM9-A* allele (A-type). Alleles to the right of the diagonal line are alleles with binding sequences more similar to the *PRDM9-C* allele (C-type). Note that the *L13* allele (found in one YRI child) and the *Av:0053* (N) allele are included here. By this measure, *M12*, *M15, and M21* are *A*-type alleles, and *M29* is a *C*-type allele despite clustering with *C-type* alleles or *A*-type alleles in panel **(A)**, respectively. **(B)** Alleles found in the populations from this study. The size of each circle indicates the number of alleles. **(C)** All human *PRDM9* alleles including blood-/sperm-only variants. **(D)** Among *PRDM9* alleles found in our study, *A*-type alleles are significantly shorter (median = 13 ZFs) than *C*-type alleles (median = 15 ZFs) (*P* = 10^–5^; Wilcoxon test). **(E)** *PRDM9* variant alleles that arise in human sperm remain a similar size to the parental alleles. All sperm variants in individuals homozygous for *A*-type/*C*-type alleles of the same length were used. **(F)** Most variant alleles in sperm retain the parental *PRDM9* binding site.

Supplementary Figure 5 *PRDM9* genotype and allele distribution by population. **(A)** The percentage (left; red) and count (right; blue) of each *PRDM9* allele in all populations. *A*-type alleles are labeled magenta and *C*-type are green. **(B)** The percentage (left; red) and count (right; blue) of individuals with at least a single copy of each *PRDM9* allele in all populations. **(C)** The count of diploid *PRDM9* genotypes in all populations.

Supplementary Figure 6 Comparison of allele frequencies between populations. Our estimates of *PRDM9* allele frequencies are susceptible to substantial sampling error as the number of alleles is high compared to the number of individuals assessed. To facilitate cross-comparison of the population frequencies of *PRDM9* alleles, we estimated the effects of sampling noise. For each population, we performed 10,000 bootstrapped samplings of alleles in the population. For each iteration, we randomly selected *N* alleles (*N* = number of alleles detected in the true population); selection was weighted by the observed allele frequency in the population. The 99% confidence intervals of each distribution are shown. Only alleles where the value of the 1st percentile is >0% in one population are shown (i.e., the estimated likelihood of 0 observations in the population is <1%). Bars show the observed frequency of each allele.

Supplementary Figure 7 The prevalence of associated SNPs among populations. Assessment of the prevalence of SNPs associated with *PRDM9* alleles split by population. Individuals were classified as homozygous (HOM), heterozygous (HET), or non-carriers (NONE) of the *PRDM9* allele indicated in gray in the column header. The prevalence of both alleles of each SNP was assessed in each group. Larger circle size and deeper red color indicate a higher prevalence.

Supplementary Figure 8 Templating errors as a source of *PRDM9* variation. The *PRDM9* ZF array is composed of tandem copies of highly similar C_2_H_2_ ZF domains. Each domain is 84-bp long, and most domains differ from each other by just 1–4 bp. This structure has the potential to cause templating errors during DNA transactions such as DNA replication or recombination. **(A)** One potential mechanism by which template switching can generate new *PRDM9* alleles. The identical sections of C_2_H_2_ ZFs are represented as black lines; colored boxes represent variable regions. For illustrative purposes, these regions are depicted as disproportionately large (depiction is ∼10 × wider) and in the context of replication. Replication pausing/slippage coupled with secondary structure formation has the potential to cause template switches during replication. (Left) Intramolecular reactions on the replicated strand can result in the duplication of ZF domains in the replicated DNA. (Right) Intramolecular reactions on the template strand can result in the deletion of ZF domains in the replicated DNA (note that the decision to depict secondary structures as loops is arbitrary). This is one mechanism by which new alleles may arise and is intended to be illustrative; however, numerous other template switching interactions may play a role. **(B,C)** Putative template switching events that give rise to one allele from another are inferred computationally. **(B)** A single template switch within or between *PRDM9-A* alleles can give rise to the *PRDM9-N* allele. The ZF codes for each allele are shown on top. The :F:I ZFs in *PRDM9-A* are replaced with a | d ZF in *PRDM9-N*. These three ZFs differ between each other at two nucleotide positions [comparison shown as the Compact Idiosyncratic Gapped Alignment Report (CIGAR) format; Li et al., 2009]. In subsequent panels, for simplicity, we drop the non-alphanumeric first character for each ZF code. Although the ZF codes are depicted in this figure, our algorithm operates on the DNA sequences. This allows us to capture template switches that create hybrid new ZFs from a combination of parental ZFs. A case in point is the | d ZF, which can be created by a template switch from after the first variable residue in :F to before the last variable residue in :I. Our algorithm first identifies the longest common 5′ subsequence between one parent allele and the putative progeny allele **(1)**. We then remove this matched sequence from the child sequence **(2)** and find the longest possible perfect match to the 5′ end of the second parental allele. **(3)** This represents a template switch event. In the case of the *PRDM9-N* allele, a perfect match is found by a single template switch that skips a single ZF (half of :F and half of :I). This process is re-iterated until either the entire progeny sequence has been matched or until no further match can be found **(4)**. In the latter case, we conclude that parentage cannot be inferred. In the former case, we can infer putative parentage. The inferred events are shown in the result panel (TS = template switch) for either a mono-allelic template switch [where the TS arises like in panel **(A)**] or for a bi-allelic TS that involves both parental alleles. **(C)** Two template switches within or between *PRDM9-C* alleles can give rise to the *PRDM9-L4* allele. This example demonstrates how our iterative algorithm captures these events (steps 2 and 3 are repeated).

Supplementary Figure 9 Most *PRDM9* variants that arise in blood and sperm can be generated from template switching between parental alleles. We analyzed all *PRDM9* allelic variants from blood and sperm that were identified in Jeffreys et al. (2013) to determine if each allele could be generated *via* template switching between parental alleles (see section “Materials and Methods”). Each *PRDM9* allele is illustrated as a series of connected boxes, where each box represents a single ZF. Alleles are grouped by the man in which they were identified (indicated in gray boxes along with the man’s diploid *PRDM9* genotype; S, sperm and B, blood). If several combinations of parental alleles are possible, we identified the “most-likely” recombinant as that which required the minimal number of template switches. If multiple possible combinations remain, one is randomly chosen for display. Colored circles indicate the alleles where this “most-likely” recombinant is derived from either both parental alleles (yellow: bi-parental) or where a uni- and bi-parental origin are equally possible (orange: Uni/Bi). ZFs are colored by the parent of origin (yellow = first allele; blue = second allele). Green ZFs indicate the region in which a template switch was inferred. Note that if template switches occur in adjacent ZFs, the resolution of this representation does not allow the source of the intervening DNA to be shown. It should be particularly noted at the few alleles derived from obligatory bi-parental switches that have double switch events in a short span (e.g., Man 14—*Dv:0445*). In these cases, the schematic appears to lack any segment from one parent because it is too short to be shown.

Supplementary Figure 10 Contributions of different *PRDM9* alleles to the DSB landscape across individuals. **(A)** Pairwise comparisons of DSB hotspots in all individuals. The bottom left panels show the correlation of log-transformed hotspot strength at shared hotspots. The number of shared hotspots is shown in green, and the Pearson correlation coefficient of log-transformed strength is shown in red. The 4–6% of *C/L4* hotspots shared with the *A/A*, *A/B*, and *A/N* individuals are likely chance overlaps. The top-right panels depict the number of hotspots in each comparison that are shared by both individuals (both: gray) or that are unique to either parent (purple and orange). The area of each rectangle represents the number of hotspots. **(B)** The hotspot count per individual. **(C)** The distribution of Pearson correlation coefficients for each sample. **(D)** The total number of unique hotspots per individual (expressed as the percent of all hotspots). **(E)** Percent of unique hotspots in the top 13,373 hotspots (by strength) for each sample. In the smallest sample, the number of hotspots is13,373 (*C/L4*). Normalizing the number of hotspots helps to control for weak and apparently unique hotspots that are only found in better samples. **(F)** Hotspots split by the likely defining allele of *PRDM9* in each individual. *PRDM9-A*-defined hotspots were those found in any of the *A/A* individuals and not in the *C/L4* individual. *B*-, *C*-, and *N*-defined hotspots were the non-*A*-defined hotspots in the respective heterozygous individuals. *PRDM9-L4*-defined hotspots were those in the *C/L4* individual that were not found in the *A/C* individual. Hotspots that match two of these criteria were designated as ambiguous (X: gray). **(G)** The contribution of each *PRDM9* allele to hotspot strength. **(H)** Some alleles of *PRDM9* define stronger hotspots in heterozygous individuals. Ambiguous hotspots are not shown. Because of differences in the numbers of hotspots, values should not be compared across individuals.

Supplementary File 1 Details of *PRDM9* genotypes for all individuals.

Supplementary File 2 Details and nomenclature for human *PRDM9* zinc fingers.

Supplementary File 3 Details of human *PRDM9* alleles.

Supplementary File 4 Details of SNP association analyses.
