# Supplementary figures and images for: Cataloging Human PRDM9 Allelic Variation Using Long-Read Sequencing Reveals PRDM9 Population Specificity and Two Distinct Groupings of Related Alleles
Source: Front Cell Dev Biol. 2021 Nov 4;9:675286. doi: 10.3389/fcell.2021.675286 (PMC8600002; doi:10.3389/fcell.2021.675286)

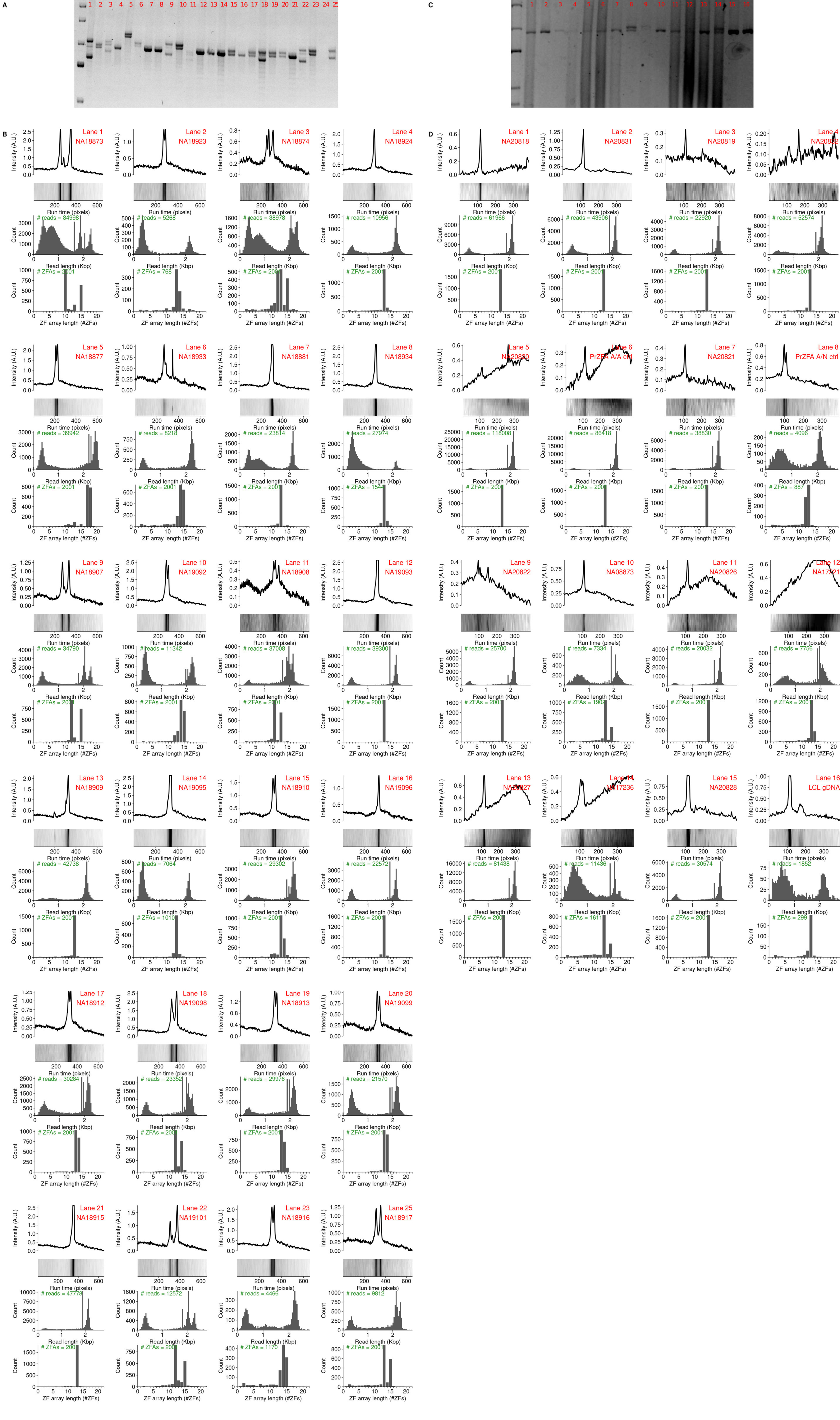

Supplement: Supplementary Figure 1 — PCR amplification biases are removed in silico. Agarose gels for post-PCR amplified PRDM9 ZF arrays. Raw gel images are shown in (A,C). Per-lane quantification is shown in (B,D). Pixels used for quantification are shown beneath each histogram. Gel quantification was performed using the R imager package. For each sample/lane, the middle plot depicts the read length distribution. The lower plot depicts the inferred lengths of PRDM9 ZF arrays from all sequencing reads used for final genotyping. For all individuals with sufficient coverage, we observe either one or two peaks in PRDM9 ZF array length. PRDM9 ZF arrays that do not coincide with the major peaks likely represent PCR artifacts that serendipitously created an erroneous, but complete, ZF array. These are a small minority of reads for all individuals. Coverage above 2,001 reads is not considered. (A,B) Twenty-four individuals with varying degrees of “laddering.” (C,D) Sixteen individuals with various amplification issues. Lane 2: cleanly amplified product. Lane 4: little/no amplification. Lane 12: smearing (unknown reason). DNA from lanes 4, 5, 9, and 12 were not used for sequencing. Further amplification experiments were performed and used for sequencing. The LCL gDNA control was DNA from an LCL cell line used as an amplification control. Lanes 6 and 8 contain DNA amplified from an A/A and A/N individual, respectively. [file Image_1.JPEG]

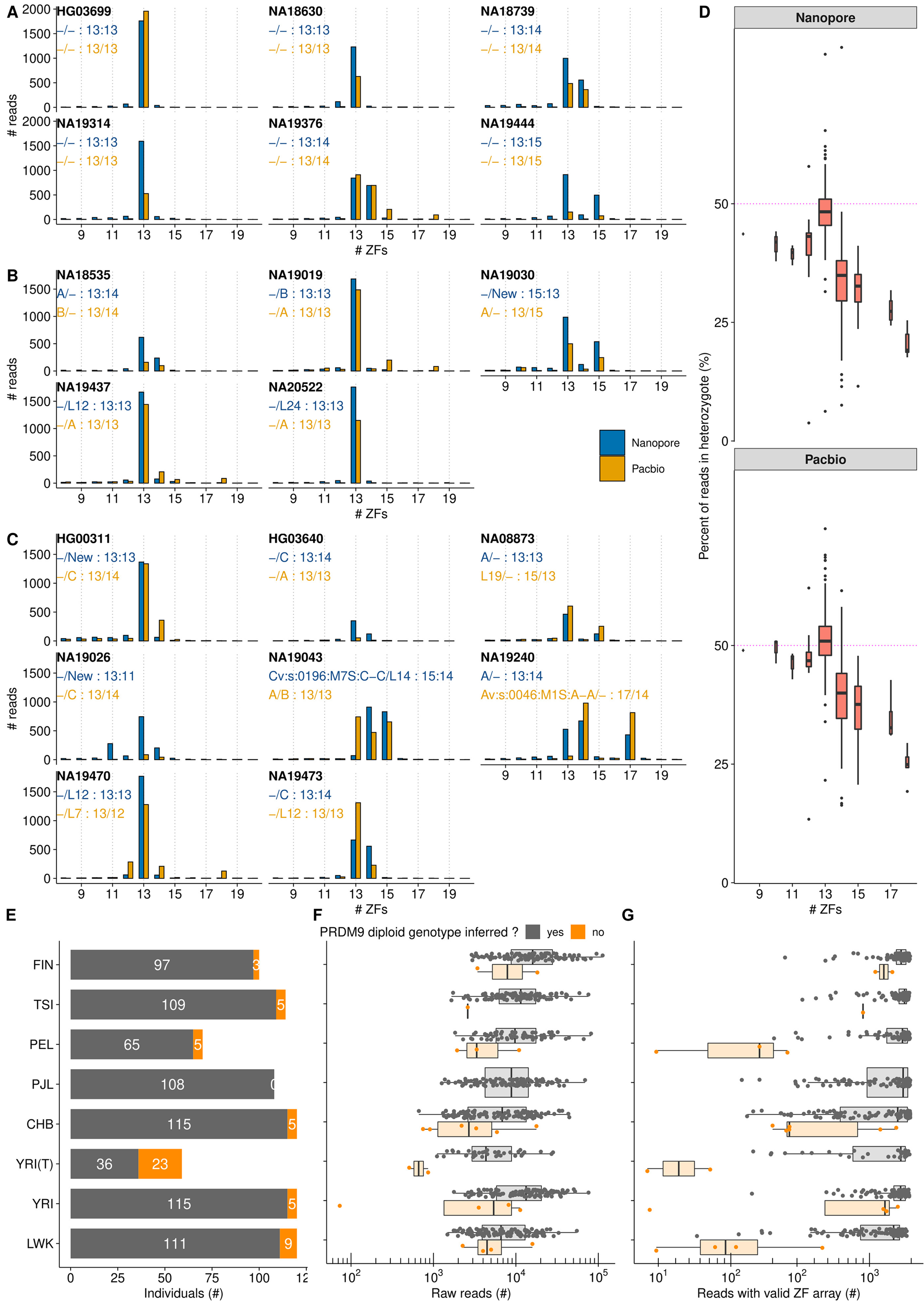

Supplement: Supplementary Figure 2 — The diploid PRDM9 genotype can be inferred from long-read sequencing data for most individuals. (A–C) Inferred ZF array sizes (in # of ZFs) using PacBio or Nanopore reads. Each individual ID is shown in black. The inferred genotypes from Nanopore (blue) and PacBio (orange) are shown alongside the inferred diploid ZF sizes. Concordant alleles are replaced with hyphens (–). New alleles are not named, as the nomenclature is determined after pooled genotyping. (A) Six representative individuals with concordant genotypes. (B) Five individuals had discordant genotypes where the number of ZFs matched. Three out of five are the result of differences at a single nucleotide (NA18535, NA19019, and NA19030). For the other two individuals, the discordant alleles differ by 3 nt (NA20522; A vs. L24 allele) and 21 nt (NA19437; A vs. L12 allele). (C) Differing ZF lengths were the cause of the eight remaining discordant genotypes. This mostly occurs because of insufficient sequencing depth or under-representation of an allele with one technology (HG03640, NA08873, NA19026, and NA19473). In three cases, the amplicon size distribution differed between the reads from the two technologies (HG00311, NA19240, and NA19043). For the remaining individual (NA19470), it appears that a 12-ZF amplicon was erroneously considered a valid allele for PacBio. (D) Detection bias for shorter PRDM9 alleles. For all heterozygous individuals, the percentage of reads for each genotype was calculated. These percentages are shown for alleles of different sizes. Absent any bias, all boxplots should have a median at 50%. The reduced coverage for longer alleles is seen for both technologies and therefore is most likely derived from the PCR step during library preparation. Thirteen-ZF alleles have apparently higher coverage; however, this is likely because most heterozygotes with one long allele also contain a 13-ZF allele (i.e., PRDM9-A). In these individuals, the relative amount of 13-ZF sequences will often be [file Image_2.JPEG]

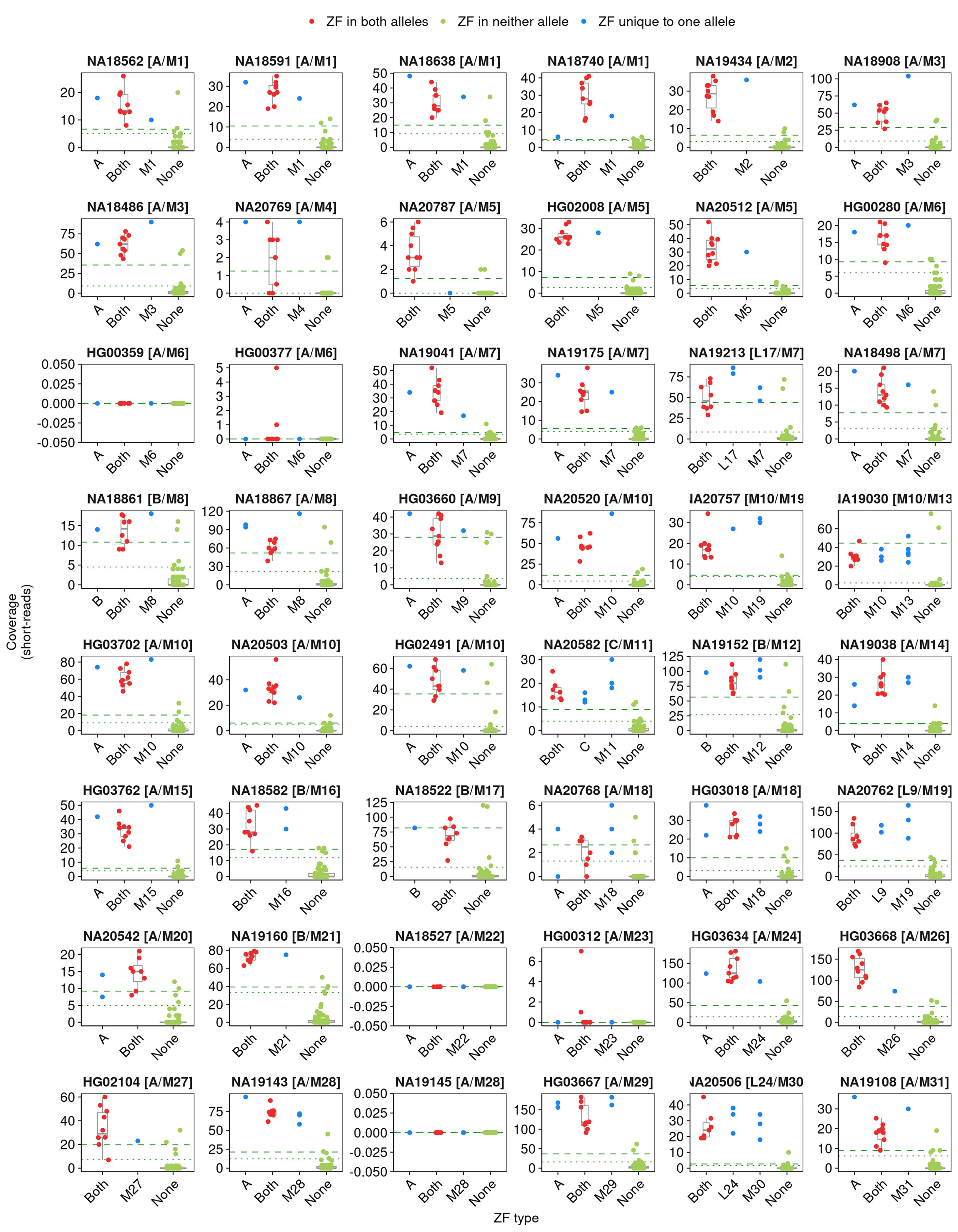

Supplement: Supplementary Figure 3 — Most novel alleles are validated using exome sequencing data. We extracted exome sequencing reads that mapped to the PRDM9 ZF array (1000 Genomes Project), then aligned these to all possible PRDM9 ZFs (see section “Materials and Methods”) for each individual. Short-read exome sequencing data were available, and analyses are presented here for 48/53 individuals who have at least one previously unannotated PRDM9 allele. Most exome sequencing was performed with 75-bp reads. Reads aligned with <72 bp or with mismatches were discarded. Since each ZF is 84-bp long, some reads may still map ambiguously despite these stringent criteria. We compared read density for ZFs found in both alleles (red), ZFs found uniquely in each allele (blue), and ZFs that were not found in either allele (green). The 95th and 98th percentile for ZFs not found in either allele are indicated with green dotted and dashed lines, respectively. Coverage is normalized for the number of each ZF present in an individual; coverage for ZFs not in the individual were normalized by 1. For three individuals, no reads passing our criteria were identified (NA19145, NA18527, and HG00359), and for five further individuals (HG00377, HG00312, NA20787, NA20768, and NA20769), very few reads were identified. For all remaining individuals, normalized read coverage for the novel allele(s) exceeded the 95th percentile of coverage for the control alleles. [file Image_3.JPEG]

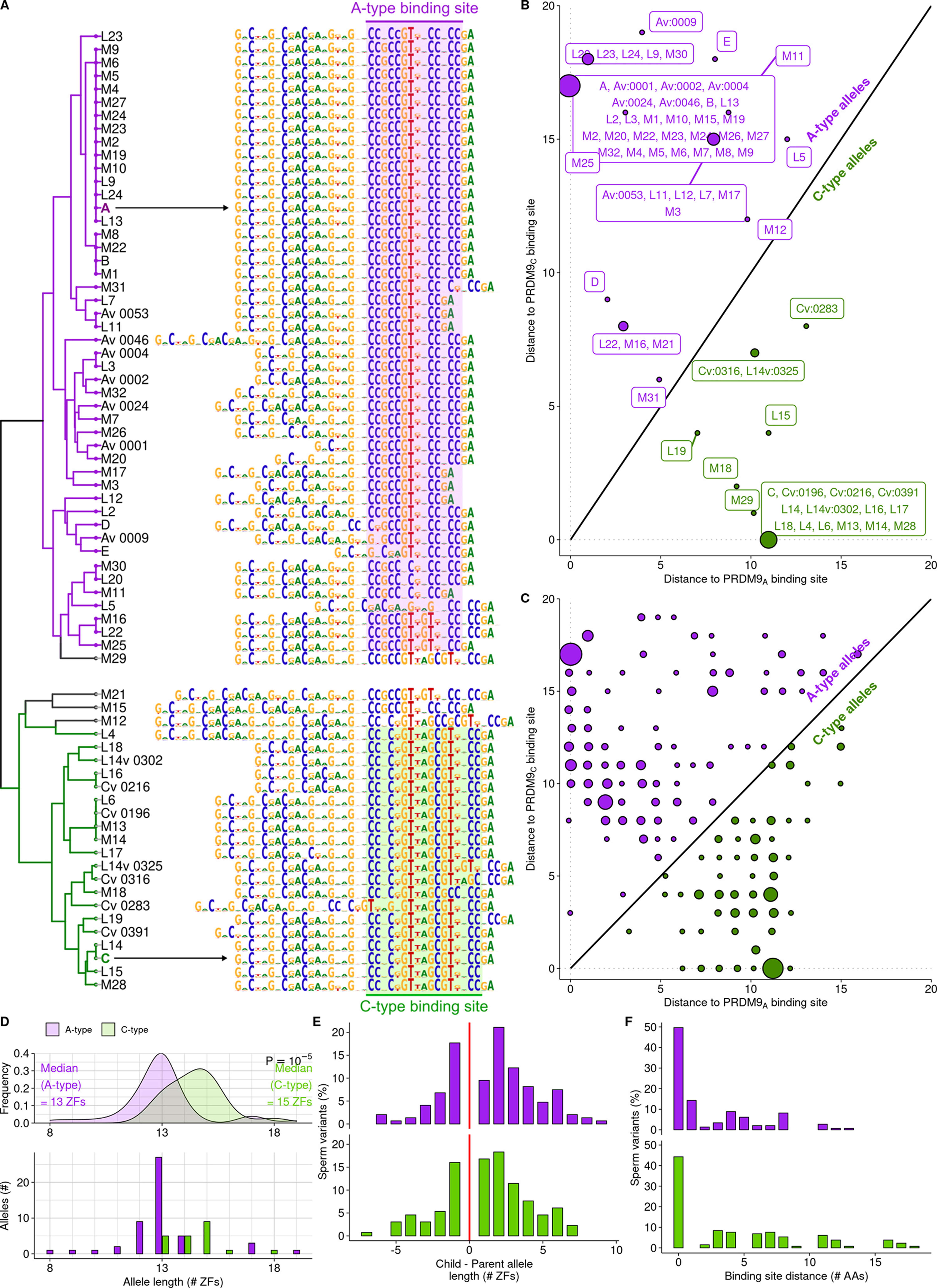

Supplement: Supplementary Figure 4 — PRDM9 alleles are categorized as A-type and C-type. (A) PRDM9 alleles found in human populations broadly cluster into two groups defined by similarity to the PRDM9-A or PRDM9-C binding site. The binding site for each allele was predicted (Persikov and Singh, 2014), and alleles were clustered using the position weight matrix for each allele (motifStack function of R motifPiles library). The regions matching the known binding residues for PRDM9-A (purple) and PRDM9-C (green) are highlighted. Motifs were manually aligned to highlight these loci. (B,C) A distance metric that measures the similarity to the amino acid sequence that defines PRDM9-A and PRDM9-C binding (distance = # of mismatches + # of gaps from a BLAST alignment) was used. Alleles left of the diagonal line are alleles with binding sequences more similar to the PRDM9-A allele (A-type). Alleles to the right of the diagonal line are alleles with binding sequences more similar to the PRDM9-C allele (C-type). Note that the L13 allele (found in one YRI child) and the Av:0053 (N) allele are included here. By this measure, M12, M15, and M21 are A-type alleles, and M29 is a C-type allele despite clustering with C-type alleles or A-type alleles in panel (A), respectively. (B) Alleles found in the populations from this study. The size of each circle indicates the number of alleles. (C) All human PRDM9 alleles including blood-/sperm-only variants. (D) Among PRDM9 alleles found in our study, A-type alleles are significantly shorter (median = 13 ZFs) than C-type alleles (median = 15 ZFs) (P = 10–5; Wilcoxon test). (E) PRDM9 variant alleles that arise in human sperm remain a similar size to the parental alleles. All sperm variants in individuals homozygous for A-type/C-type alleles of the same length were used. (F) Most variant alleles in sperm retain the parental PRDM9 binding site. [file Image_4.JPEG]

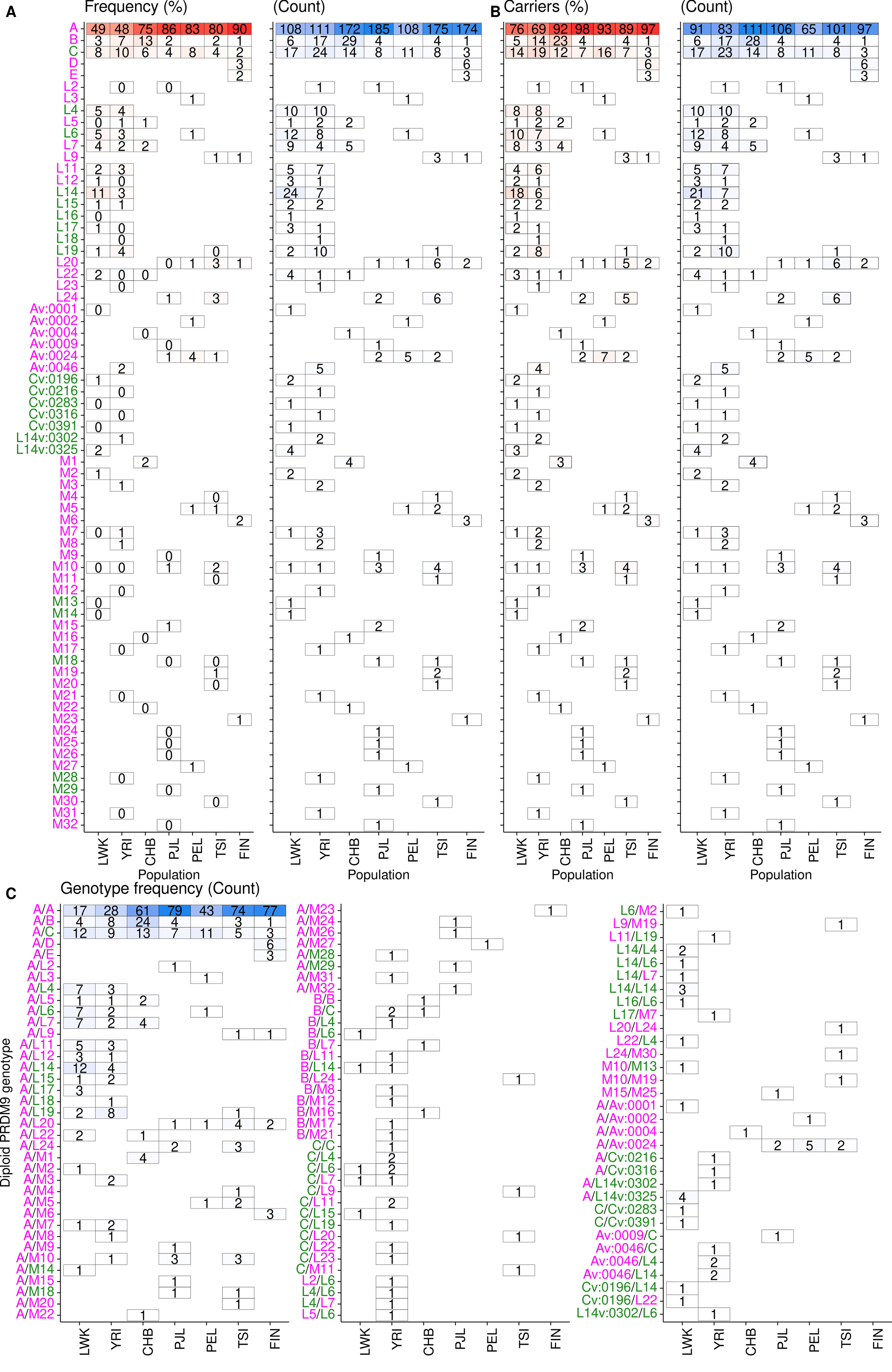

Supplement: Supplementary Figure 5 — PRDM9 genotype and allele distribution by population. (A) The percentage (left; red) and count (right; blue) of each PRDM9 allele in all populations. A-type alleles are labeled magenta and C-type are green. (B) The percentage (left; red) and count (right; blue) of individuals with at least a single copy of each PRDM9 allele in all populations. (C) The count of diploid PRDM9 genotypes in all populations. [file Image_5.JPEG]

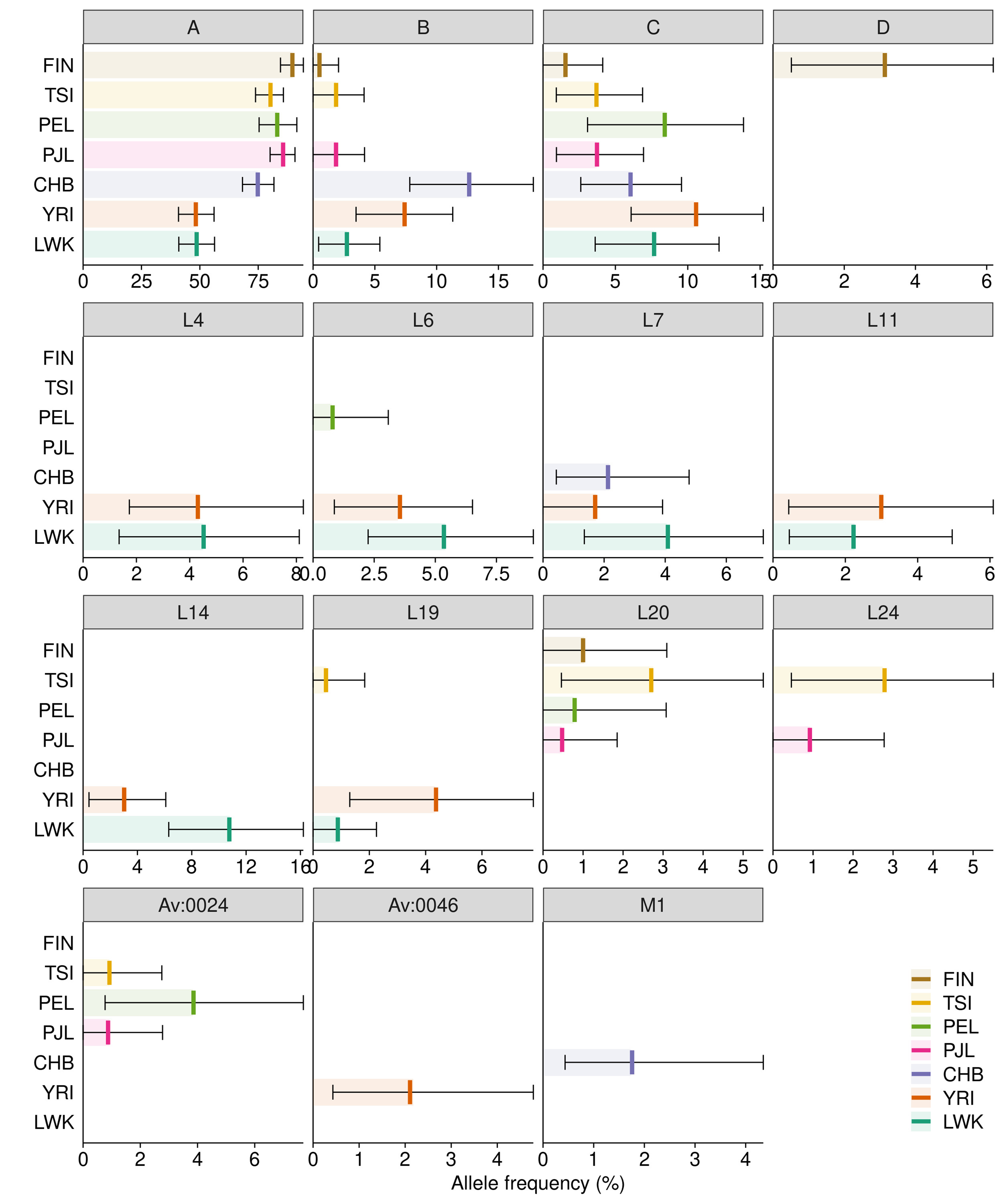

Supplement: Supplementary Figure 6 — Comparison of allele frequencies between populations. Our estimates of PRDM9 allele frequencies are susceptible to substantial sampling error as the number of alleles is high compared to the number of individuals assessed. To facilitate cross-comparison of the population frequencies of PRDM9 alleles, we estimated the effects of sampling noise. For each population, we performed 10,000 bootstrapped samplings of alleles in the population. For each iteration, we randomly selected N alleles (N = number of alleles detected in the true population); selection was weighted by the observed allele frequency in the population. The 99% confidence intervals of each distribution are shown. Only alleles where the value of the 1st percentile is >0% in one population are shown (i.e., the estimated likelihood of 0 observations in the population is <1%). Bars show the observed frequency of each allele. [file Image_6.JPEG]

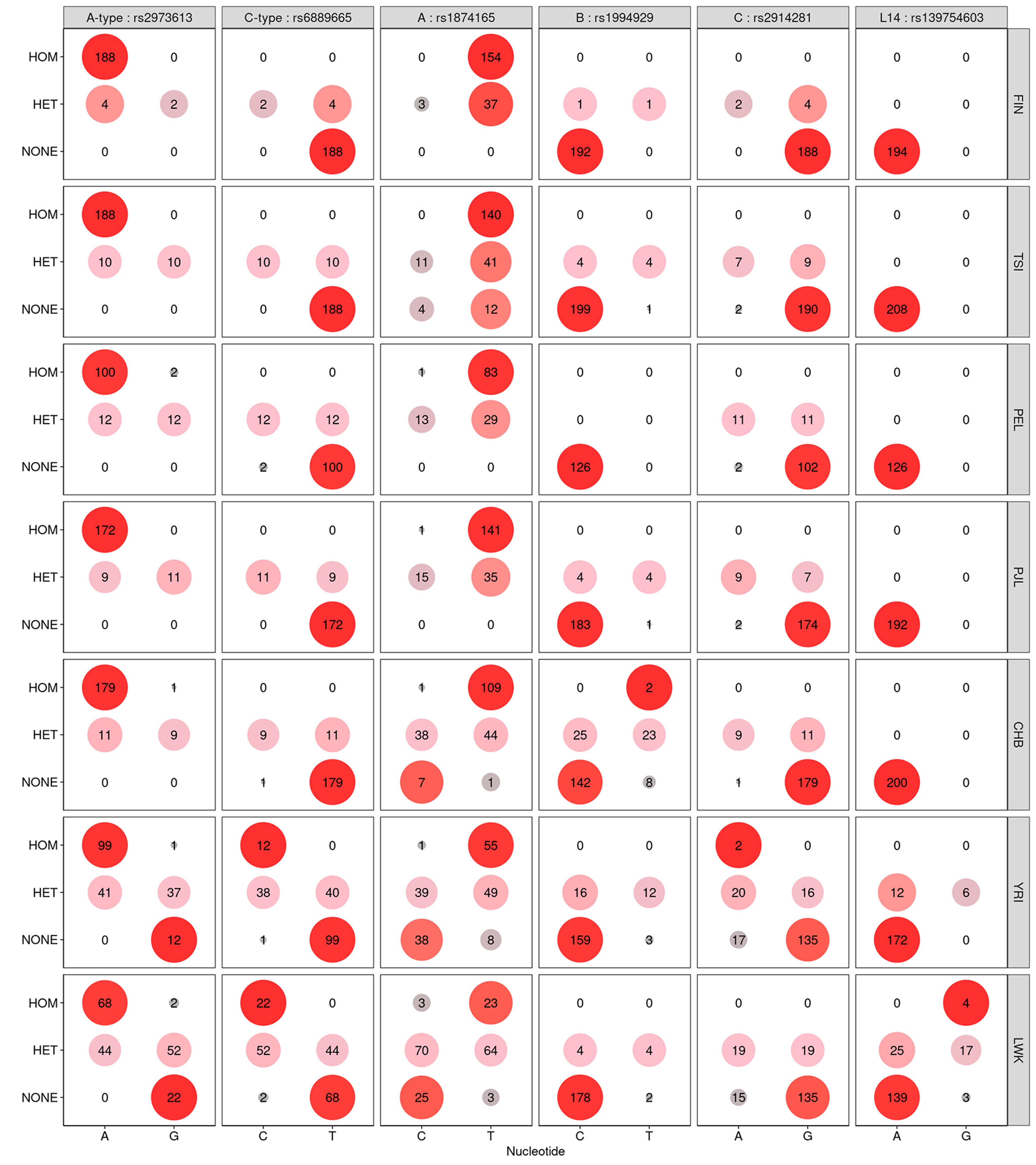

Supplement: Supplementary Figure 7 — The prevalence of associated SNPs among populations. Assessment of the prevalence of SNPs associated with PRDM9 alleles split by population. Individuals were classified as homozygous (HOM), heterozygous (HET), or non-carriers (NONE) of the PRDM9 allele indicated in gray in the column header. The prevalence of both alleles of each SNP was assessed in each group. Larger circle size and deeper red color indicate a higher prevalence. [file Image_7.JPEG]

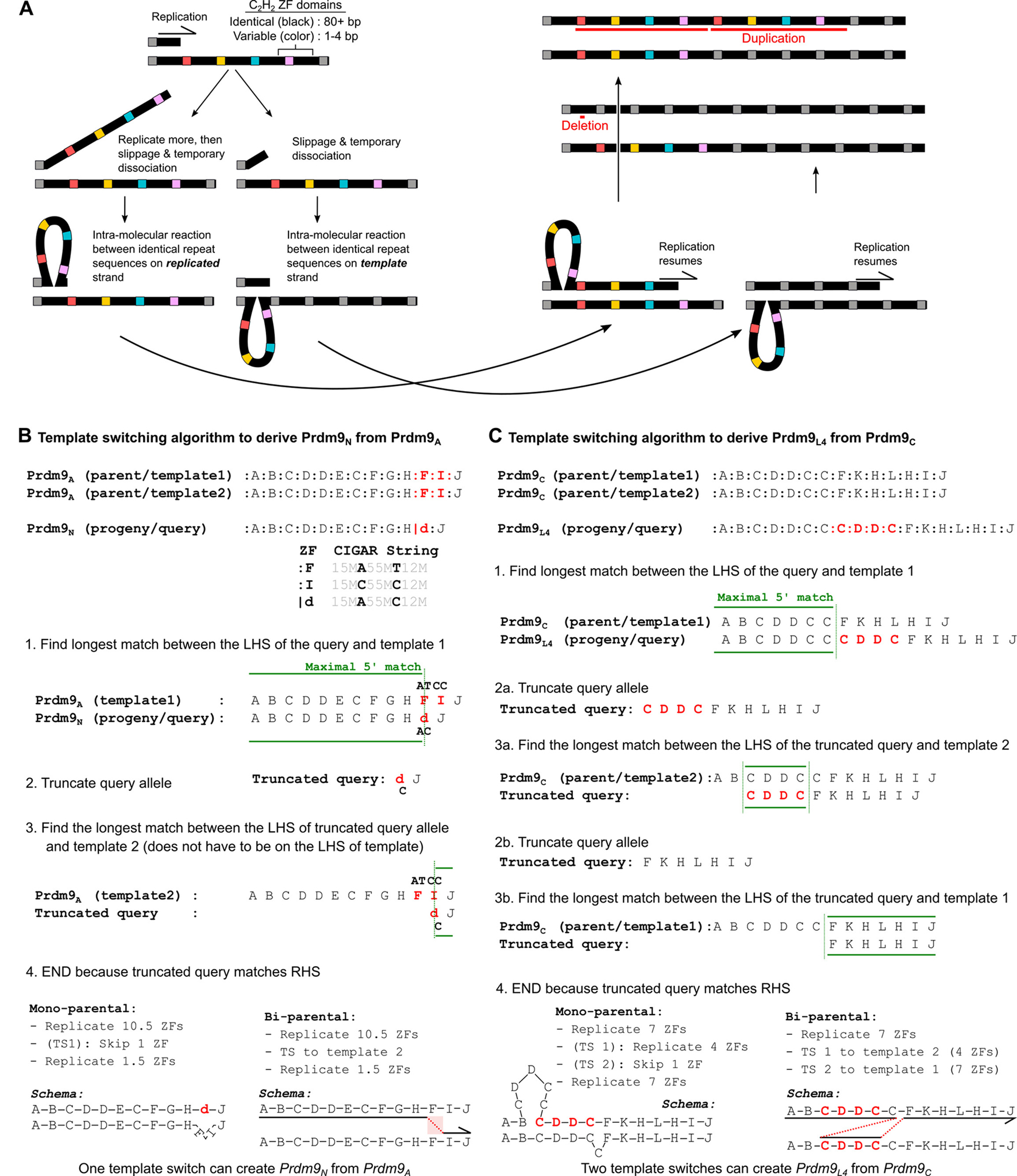

Supplement: Supplementary Figure 8 — Templating errors as a source of PRDM9 variation. The PRDM9 ZF array is composed of tandem copies of highly similar C2H2 ZF domains. Each domain is 84-bp long, and most domains differ from each other by just 1–4 bp. This structure has the potential to cause templating errors during DNA transactions such as DNA replication or recombination. (A) One potential mechanism by which template switching can generate new PRDM9 alleles. The identical sections of C2H2 ZFs are represented as black lines; colored boxes represent variable regions. For illustrative purposes, these regions are depicted as disproportionately large (depiction is ∼10 × wider) and in the context of replication. Replication pausing/slippage coupled with secondary structure formation has the potential to cause template switches during replication. (Left) Intramolecular reactions on the replicated strand can result in the duplication of ZF domains in the replicated DNA. (Right) Intramolecular reactions on the template strand can result in the deletion of ZF domains in the replicated DNA (note that the decision to depict secondary structures as loops is arbitrary). This is one mechanism by which new alleles may arise and is intended to be illustrative; however, numerous other template switching interactions may play a role. (B,C) Putative template switching events that give rise to one allele from another are inferred computationally. (B) A single template switch within or between PRDM9-A alleles can give rise to the PRDM9-N allele. The ZF codes for each allele are shown on top. The :F:I ZFs in PRDM9-A are replaced with a | d ZF in PRDM9-N. These three ZFs differ between each other at two nucleotide positions [comparison shown as the Compact Idiosyncratic Gapped Alignment Report (CIGAR) format; Li et al., 2009]. In subsequent panels, for simplicity, we drop the non-alphanumeric first character for each ZF code. Although the ZF codes are depicted in this figure, our algorithm operates on the DNA sequences. T [file Image_8.JPEG]

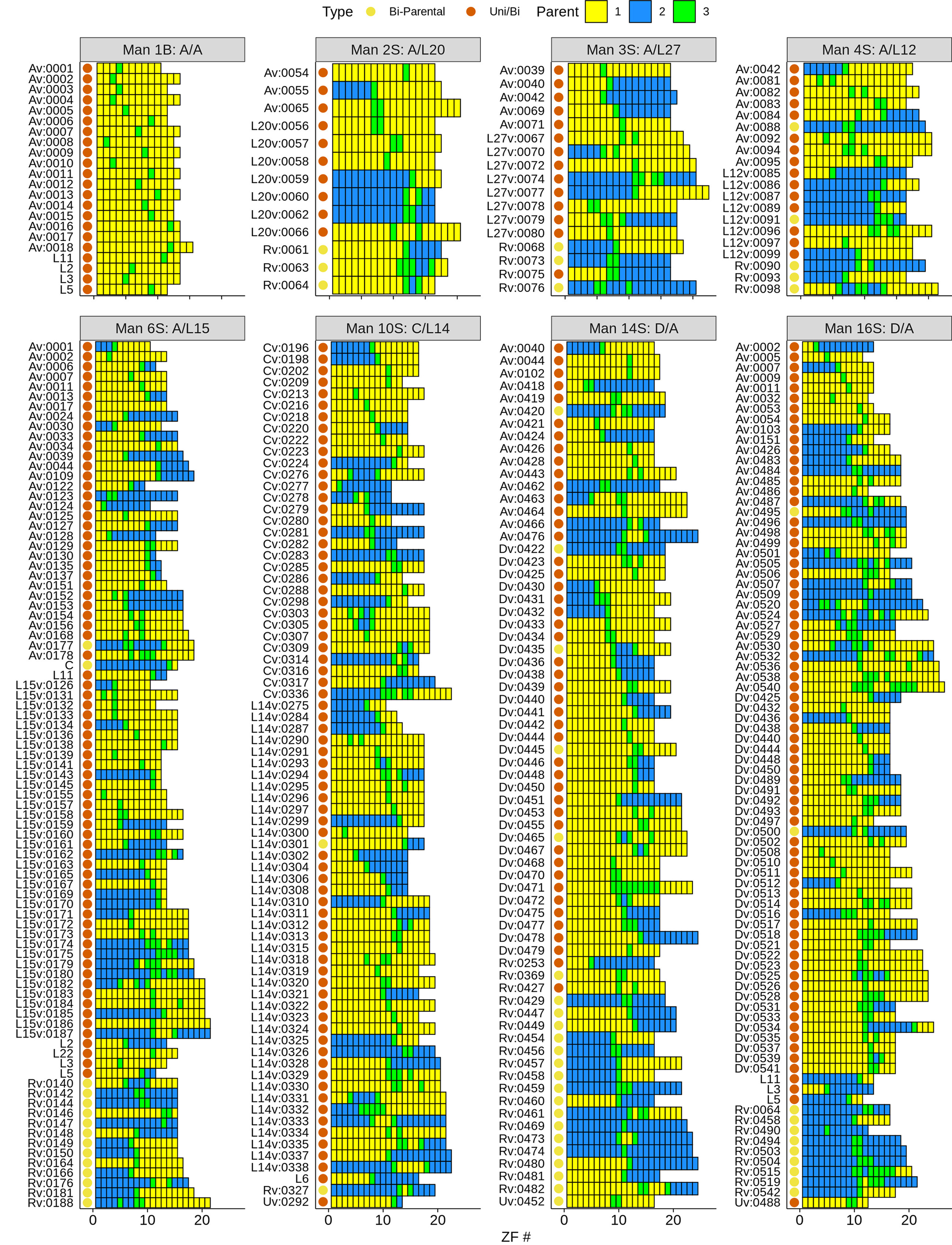

Supplement: Supplementary Figure 9 — Most PRDM9 variants that arise in blood and sperm can be generated from template switching between parental alleles. We analyzed all PRDM9 allelic variants from blood and sperm that were identified in Jeffreys et al. (2013) to determine if each allele could be generated via template switching between parental alleles (see section “Materials and Methods”). Each PRDM9 allele is illustrated as a series of connected boxes, where each box represents a single ZF. Alleles are grouped by the man in which they were identified (indicated in gray boxes along with the man’s diploid PRDM9 genotype; S, sperm and B, blood). If several combinations of parental alleles are possible, we identified the “most-likely” recombinant as that which required the minimal number of template switches. If multiple possible combinations remain, one is randomly chosen for display. Colored circles indicate the alleles where this “most-likely” recombinant is derived from either both parental alleles (yellow: bi-parental) or where a uni- and bi-parental origin are equally possible (orange: Uni/Bi). ZFs are colored by the parent of origin (yellow = first allele; blue = second allele). Green ZFs indicate the region in which a template switch was inferred. Note that if template switches occur in adjacent ZFs, the resolution of this representation does not allow the source of the intervening DNA to be shown. It should be particularly noted at the few alleles derived from obligatory bi-parental switches that have double switch events in a short span (e.g., Man 14—Dv:0445). In these cases, the schematic appears to lack any segment from one parent because it is too short to be shown. [file Image_9.JPEG]

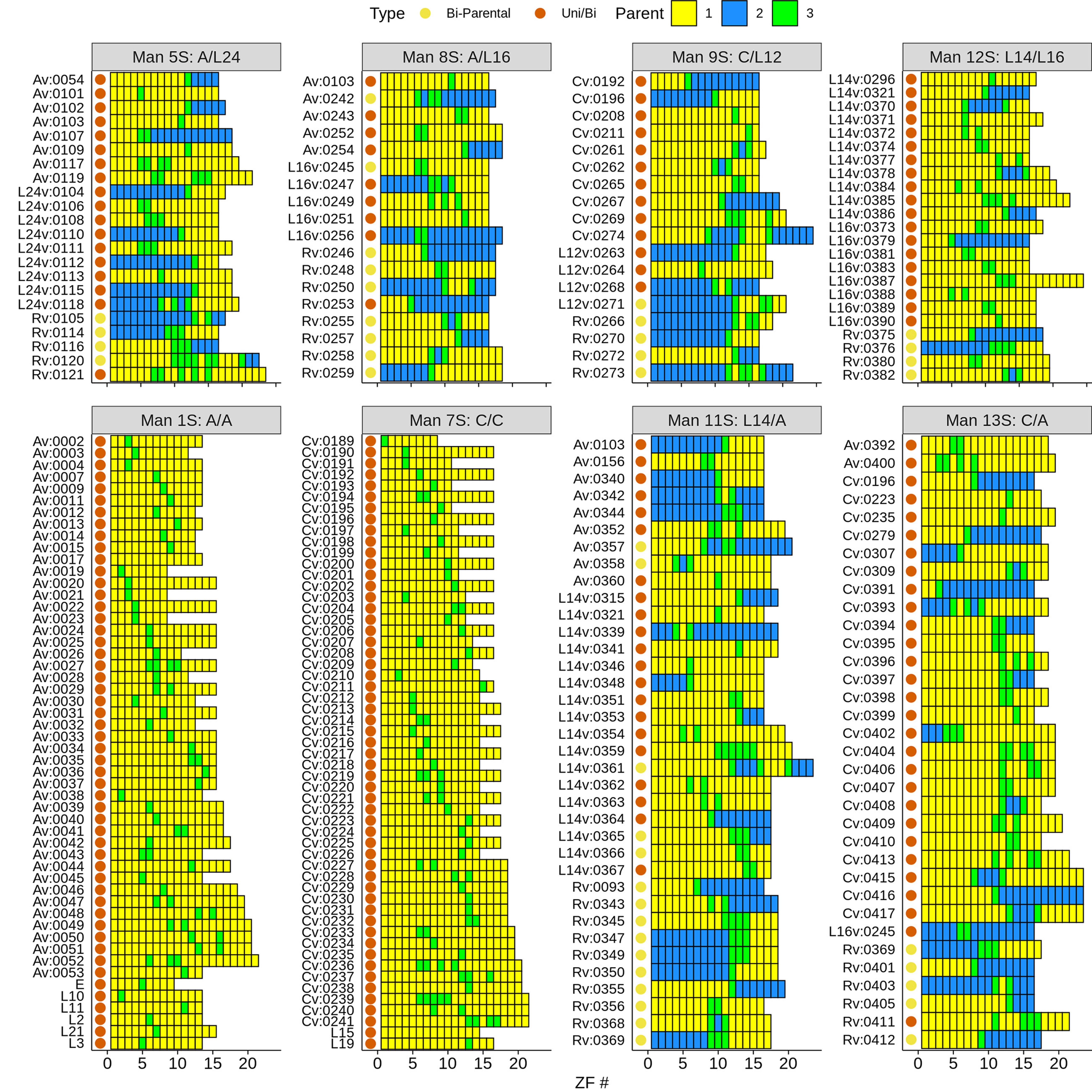

Supplement: Supplementary Figure 10 — Contributions of different PRDM9 alleles to the DSB landscape across individuals. (A) Pairwise comparisons of DSB hotspots in all individuals. The bottom left panels show the correlation of log-transformed hotspot strength at shared hotspots. The number of shared hotspots is shown in green, and the Pearson correlation coefficient of log-transformed strength is shown in red. The 4–6% of C/L4 hotspots shared with the A/A, A/B, and A/N individuals are likely chance overlaps. The top-right panels depict the number of hotspots in each comparison that are shared by both individuals (both: gray) or that are unique to either parent (purple and orange). The area of each rectangle represents the number of hotspots. (B) The hotspot count per individual. (C) The distribution of Pearson correlation coefficients for each sample. (D) The total number of unique hotspots per individual (expressed as the percent of all hotspots). (E) Percent of unique hotspots in the top 13,373 hotspots (by strength) for each sample. In the smallest sample, the number of hotspots is13,373 (C/L4). Normalizing the number of hotspots helps to control for weak and apparently unique hotspots that are only found in better samples. (F) Hotspots split by the likely defining allele of PRDM9 in each individual. PRDM9-A-defined hotspots were those found in any of the A/A individuals and not in the C/L4 individual. B-, C-, and N-defined hotspots were the non-A-defined hotspots in the respective heterozygous individuals. PRDM9-L4-defined hotspots were those in the C/L4 individual that were not found in the A/C individual. Hotspots that match two of these criteria were designated as ambiguous (X: gray). (G) The contribution of each PRDM9 allele to hotspot strength. (H) Some alleles of PRDM9 define stronger hotspots in heterozygous individuals. Ambiguous hotspots are not shown. Because of differences in the numbers of hotspots, values should not be compared across individuals. [file Image_10.JPEG]

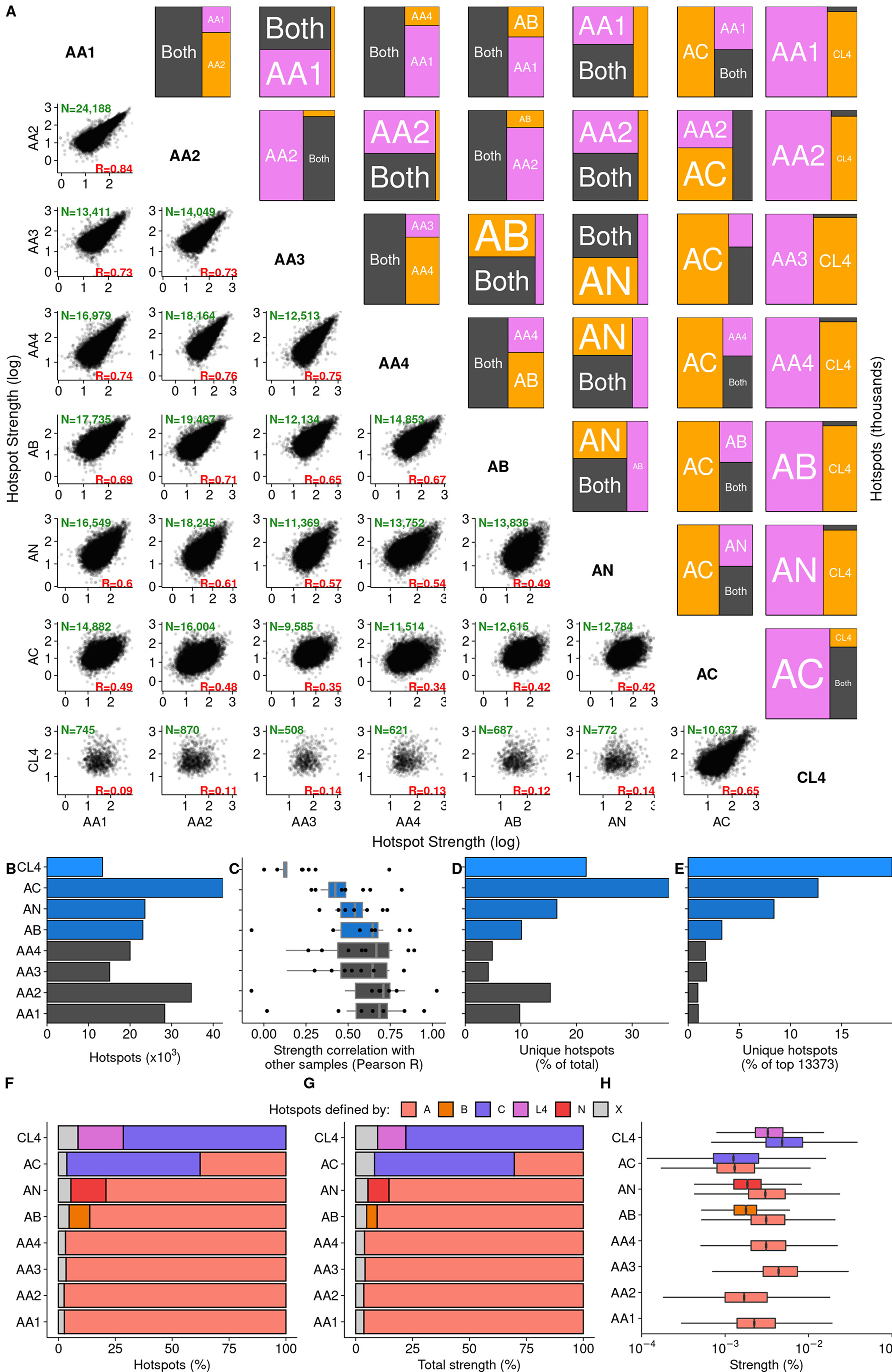

Supplement: Supplementary file 11 [file Image_11.JPEG]
